# Maize synthesized benzoxazinoids affect the host associated microbiome

**DOI:** 10.1186/s40168-019-0677-7

**Published:** 2019-04-11

**Authors:** Enoch Narh Kudjordjie, Rumakanta Sapkota, Stine K. Steffensen, Inge S. Fomsgaard, Mogens Nicolaisen

**Affiliations:** Faculty of Science and Technology, Department of Agroecology, Aarhus University, Forsøgsvej 1, 4200 Slagelse, Denmark

**Keywords:** Benzoxazinoids, Plant microbiome, Root exudates, Secondary metabolites, Knockout mutants, Maize microbiome

## Abstract

**Background:**

Plants actively shape their associated microbial communities by synthesizing bio-active substances. Plant secondary metabolites are known for their signaling and plant defense functions, yet little is known about their overall effect on the plant microbiome. In this work, we studied the effects of benzoxazinoids (BXs), a group of secondary metabolites present in maize, on the host-associated microbial structure. Using BX knock-out mutants and their W22 parental lines, we employed 16S and ITS2 rRNA gene amplicon analysis to characterize the maize microbiome at early growth stages.

**Results:**

Rhizo-box experiment showed that BXs affected microbial communities not only in roots and shoots, but also in the rhizosphere. Fungal richness in roots was more affected by BXs than root bacterial richness. Maize genotype (BX mutants and their parental lines) as well as plant age explained both fungal and bacterial community structure. Genotypic effect on microbial communities was stronger in roots than in rhizosphere. Diverse, but specific, microbial taxa were affected by BX in both roots and shoots, for instance, many plant pathogens were negatively correlated to BX content. In addition, a co-occurrence analysis of the root microbiome revealed that BXs affected specific groups of the microbiome.

**Conclusions:**

This study provides insights into the role of BXs for microbial community assembly in the rhizosphere and in roots and shoots. Coupling the quantification of BX metabolites with bacterial and fungal communities, we were able to suggest a gatekeeper role of BX by showing its correlation with specific microbial taxa and thus providing insights into effects on specific fungal and bacterial taxa in maize roots and shoots. Root microbial co-occurrence networks revealed that BXs affect specific microbial clusters.

**Electronic supplementary material:**

The online version of this article (10.1186/s40168-019-0677-7) contains supplementary material, which is available to authorized users.

## Background

Plants are in continuous contact with a huge diversity of microorganisms present in their environment. Accumulating evidence supports that plants and microorganisms have co-evolved and adapted to make close associations [1]. Recent studies showed that plants actively shape their associated microbiomes [2–4]. Some members of the microbiota, including arbuscular mycorrhizal fungi (AMF) and plant growth-promoting rhizobacteria (PGPR), assist the plant in nutrient absorption, pathogen resistance, and growth promotion [5]. Conversely, other members are detrimental to plant health by causing diseases that affect overall plant yield and value. While these microorganisms are well-studied, the effects on plant health and growth of the majority of the members of the microbiome are still unknown.

Plant metabolites are considered as a major player in microbe assembling in plants, as metabolites not only support the microbial life close to the plant as source of nutrients, but also act in dynamic processes of selecting and suppressing microbes for the benefits of the plant [6]. While some effects of plant metabolites on rhizosphere colonization by microbial communities have been proposed, only a relatively small number of metabolites that are essential for microbial assembly are presently known. Flavonoids, for instance, are known to mediate positive interactions between legume roots and nitrogen-fixing bacteria [7]. Similarly, organic acids such as citric and fumaric acid have been reported to attract a plant growth-promoting *Pseudomonas fluorescens* strain to roots [8]. Glucosinolates in *Arabidopsis thaliana* have been shown to significantly influence the root and rhizosphere microbiome [3]. Badri and coworkers also found changes in the *A. thaliana* rhizosphere microbiome using ABC transporter mutants with different metabolite profiles [9]. Moreover, p-coumaric acid has been reported to affect the composition of microbial communities in the rhizosphere of cucumber [10].

Benzoxazinoids (BXs) are produced by several species of the grasses (Poaceae), mostly during the early stages of plant development, and steadily declining as the plant matures. BXs are indole-derived compounds, and they are found in plants in different forms [11]. In some maize inbred lines, the glucoside 2,4-dihydroxy-7-methoxy-2H-1,4-benzoxazin-3(4H)-one (DIMBOA-Glc) and its precursor 2,4-di-hydroxy-2H-1,4-benzoxazin-3(4H)-one (DIBOA-Glc) are predominant [12–14]. When secreted into the soil, 6-methoxy-benzoxazolin-2-one (MBOA), a DIMBOA hydrolysis product, undergoes microbial transformation to produce phenoxazinones [15], a group of compounds known for their broad and powerful biocidal properties [16]. BXs have been widely acknowledged for their importance in plant defense [17], against insect pests and pathogens in both above and below ground parts of cereals [18–21], but also in allelopathy [21, 22]. For example, BXs are antagonistic towards insects, including aphids and corn borers, the bacterial genus *Bacillus* [23], and fungi such as species of *Fusarium* [21, 24]. Recent studies indicate a potential role of BXs in attracting the plant growth-promoting bacterium *Pseudomonas putida* towards plant roots [16]. So far, several examples of BX effect on single insects, plant pathogens, and beneficial microbes have been observed; however, many aspects of the influence of BXs on microbial communities in roots and shoots are still unknown.

Analyzing how specific metabolites impact the plant microbiome is a relatively novel approach to comprehensively dissect the layers of complex interactions of microbiota in the root and shoot interface, thus allowing us to unravel the mechanisms employed by the plant to shape its associated microbiome. Plant-microbial community assemblage starts at the very early development of the plant, which in maize coincides with a higher production of BXs. This leads us to the hypothesis that BXs play a major role in the assembly of the maize-associated microbiomes in roots and shoots and in the rhizosphere.

To test our hypothesis, we analyzed microbiomes in maize mutants impaired in the BX biosynthesis pathway at different steps and compared with microbiomes in isogenic W22 background parental lines.

## Materials and methods

### Plant material

We used three maize BX knockout mutants, *bx1*, *bx2*, and *bx6*, and their near-isogenic W22 background genetic stock controls T43 and a1-m3. The parental line T43 is a color-converted W22 stock carrying r1-sc::m3, a Ds6-like insertion in the r1 locus that controls anthocyanin accumulation in aleurone and scutellar tissues [25, 26]. The *bx1* and *bx6* mutants are both derived from T43 and thus have the r1-sc::m3 mutation. The a1-m3 parental line also has a Ds insertion allele a1-m3::Ds in the A1 gene that encodes a dihydroquercetin reductase (DFR) required for anthocyanin production [27]. The *bx2* mutant is derived from a1-m3 Ds insertion lines and carries the a1-m3::Ds allele. For simplicity, we renamed the parental line a1-m3 as W22_1 and T43 as W22_2. To reflect their respective backgrounds, the mutants were also renamed as *bx1*W22_2, *bx2*W22_1, and *bx6*W22_2 (Table 1). All maize seeds used in this study were kindly provided by Prof. Georg Jander, Boyce Thompson Institute, Cornell University, USA.

**Table 1:** List of maize parental lines and their mutants in the W22 genetic background with gene insertions [18]

Name	Ds insertion lines	Genotype	Maize gene ID	Gene mutation
W22_2	T43	Parental line	T43 2008	--
*bx1* W22_2	B.W06.0775	Mutant	GRMZM2G085381	bx1::Ds
*bx6* W22_2	I.S07.0479	Mutant	GRMZM6G617209	bx6::Ds
W22_1	a1-m3	Parental line	KA08-277	-
*bx2* W22_1	I.S07.3472	Mutant	GRMZM2G085661	bx2::Ds

### Experimental design

The experiment was performed in a semi-field facility at Aarhus University, Flakkebjerg, from July to August 2016. As planting medium, we used a sandy-clay-loam field soil previously cultivated with barley from Jyndevad Research Station, Denmark. Rhizoboxes that could be easily opened, with inner dimensions of 36 cm × 18 cm × 2 cm (H × L × W), were used [28]. In the rhizoboxes, the plant growth compartment was separated from the bulk soil with a nylon mesh (30 μm) (Additional file 1: Figure S1A). We covered the glass part of rhizoboxes with black plastic to prevent algal growth. Rhizoboxes were each filled with soil and arranged on rhizobox stands in a completely randomized setup (Additional file 1: Figure S1B). For each maize genotype, we sowed five replicated rhizoboxes with two maize seeds separated by 4 cm. As negative controls, we included five non-planted rhizoboxes.

Prior to sowing, we collected soil at 3–5 cm depths from each rhizobox to represent bulk soil samples at day 0. We removed weeds and irrigated the rhizoboxes twice a week. The first sampling of root, rhizosphere, and shoot was done at 10 days after sowing (DAS). At this stage, one seedling from each rhizobox was destructively sampled by opening rhizoboxes and gently removing all root parts of the seedling followed by gently wash using distilled water. The root and shoot samples were divided into two tubes, one portion used for BX quantification and the other portion for metabarcoding. Soil attached to the roots and within the root network (ca. 2 mm) was collected to represent rhizosphere samples. Bulk soil was sampled from the region separated from roots by nylon mesh. Approximately 0.5 g and 1.2 g of rhizosphere and bulk soils, respectively, were collected from each rhizobox for analysis.

Nondestructive sampling of maize roots and shoots for metabarcoding was done at 20, 30, and 40 DAS. This was done by carefully collecting few roots from single plants to minimize disturbance of maize seedling. The upper parts of leaves were sampled as shoot compartment (Additional file 1: Figure S1A). Bulk and rhizosphere soil was collected as previously described at 10, 20, 30, and 40 DAS. In total, we collected 450 samples comprising 150 bulk soils (BS) and 100 (25 × 4) each of root, rhizosphere, and shoot samples. All collected samples were immediately frozen in liquid nitrogen with samples for BX analysis later transferred and stored at − 80 °C while those for metabarcoding were stored at − 20 °C.

### DNA extraction

Bulk and rhizosphere soils, root, and shoot samples were lyophilized for 72 h and ground using sterile steel beads in a Geno/Grinder2000 at 1500 rpm for 3 × 30 s. From the ground soil and rhizosphere samples, 250 mg was used for soil DNA extraction using the PowerLyzer™ PowerSoil® DNA Isolation Kit (Mo Bio Laboratories, Carlsbad, CA, USA) according to the manufacturer’s instructions. Root and shoot DNA was extracted using the DNeasy Plant Mini kit (Qiagen, Hilden, Germany) according to the manufacturer’s instructions. All DNA samples were stored at − 20 °C until used for PCR.

### Library preparation and amplicon sequencing

We prepared bacterial and fungal sequencing libraries from the 450 DNA samples. The bacterial V3V4 region of the 16S rRNA gene was amplified using primers S-DBact-0341-b-S-17/S-D-Bact-0785-a-A-21 [29]. For amplification of the fungal ITS2 region, fITS7 and ITS4 primers were used [30]. PCR was performed in a reaction mixture of 25 μl consisting of 1× PCR reaction buffer, 1.5 mM MgCl_2_, 0.2 mM dNTPs, 1 μM of each primer, 1 U of GoTaq Flexi polymerase (Promega Corporation, Madison, USA), and 1 μl of DNA template. Bacterial PCR was conducted in a GeneAmp PCR System 9700 thermal cycler (Thermo Fisher Scientific) and used at 94 °C for 5 min, followed by 25 cycles at 94 °C for 30 s, 55 °C for 30 s, 72 °C for 1 min, and a final elongation step at 72 °C for 10 min. Fungal libraries were prepared similarly, except that we used an annealing temperature of 57 °C as recommended [30]. Dual indexing in combination with internal barcodes was carried out to allow pooling of 450 samples. For indexing, primers including indexing tags were used in a PCR for 10 cycles, with the thermal cycler programs as described above. In addition to dual indexing, a varying number of nucleotides were added to the forward primer as internal barcodes for combining samples within each index combination [31, 32]. In total, we used 90 index combinations with five internal barcodes for 450 samples. Primer sequences including internal barcodes and the index combinations are described in supplementary information (Additional file 2). After PCR, amplicon size was confirmed by visualization in a 1.5% agarose gel using SYBR staining. PCR products were pooled, precipitated, and re-eluted as described earlier [33]. In order to extract the expected size of the amplicons (300–450 bp) and to avoid shorter reads, the pooled DNA was separated on a 1.5% agarose gel and the band of the expected size was extracted using a QIAquick Gel Extraction Kit (Qiagen). The final concentration of the amplicon library was evaluated using a spectrophotometer. Two sequencing libraries, one each for bacteria and fungi, were shipped to Eurofins MWG (Ebersberg, Germany) for sequencing on their Illumina MiSeq platform using a dual indexing strategy. All the sequence files from this study were deposited in the NCBI sequence read archive under the SRA accession number PRJNA513956.

### Chemicals for benzoxazinoid extraction and quantification

HPLC grade methanol and acetonitrile were purchased from Rathburn (Walkerburn, Scotland); MS-grade methanol, acetonitrile, and isopropanol from Fischer Scientific (Loughborough, UK); and acetic acid from Baker (Griesheim, Germany). Standards for benzoxazinoids (Additional file 1: Table S1) were either obtained as a part of an ongoing patenting process or synthesized [34, 35].

### Extraction of samples for benzoxazinoid quantification

Maize root and shoot samples were collected at day 10, immediately frozen at − 80 °C and subsequently freeze-dried. The total amount of freeze-dried plant material of each sample (ranging from 0.03 to 0.6 g) was extracted using an accelerated solvent extraction 350 system (ASE) from Dionex following the method described earlier [36]. The extract was filtered and diluted 1:1 with water before analysis.

Rhizosphere soil samples of *bx2*W22_1 and W22_1 were freeze-dried. One milliliter of 80% MeOH/0.1% acetic acid was added to 50 mg of freeze-dried rhizosphere soil; the mixture was ultrasonicated for 45 min, centrifuged at 45 rpm for 10 min, the supernatant was removed, and the extraction was repeated. The extraction was performed in duplicate. Supernatants were combined and solvent was added up to 2 ml. The supernatant was filtered and diluted 1:4 with water before analysis.

### Quantification of BXs in plant material by LC–MS/MS

The plant extracts were analyzed by LC–MS/MS using an Agilent 1100 HPLC system coupled with a 3200 QTRAP mass spectrometer (AB SCIEX, Foster City, CA), according to Jensen et al. [35]. The chromatography was performed using a 250 mm × 2 mm id 4-μm Synergi Polar RP-80A column (Phenomenex, Macclesfield, UK) with a flow rate of 200 μL/min and an injection volume of 20 μL. The temperatures of the column oven and autosampler were set at 30 and 4 °C, respectively. Two mobile phases (A: 7% acetonitrile in water and B: 78% acetonitrile in water, each containing 20 mM of acetic acid) were used in a linear gradient system. The LC method started with 16% mobile phase B 0–5 min, followed by a 10-min gradient to 40% B. Subsequently, B was further increased to 95% within 1 min and maintained at 95% for 4 min. The gradient was finally returned to the initial condition within 1 min, and the column was re-equilibrated with the initial gradient condition for 9 min before the next injection. All other LC–MS/MS parameters were as previously described [37]. For confirmation of the presence of the compounds, analysis of samples was repeated in a Synergy Fusion 250 mm × 2 mm id 4-μm RP-80A column (Phenomenex, Macclesfield, UK). Analyst Software (version 1.6.2) was used for instrument control, data acquisition, and subsequent quantifications. Quantifications were done on the basis of standard curves prepared in the range 0.39 to 200 ng ml^−1^. Data points of the standard curves were weighted according to *x*^−1^.

### Quantification of BXs in rhizosphere soil by LC–MS/MS

The rhizosphere soil extracts were analyzed by LC–MS/MS using an Agilent 1200 HPLC system coupled with a 4500 QTRAP mass spectrometer (AB SCIEX, Foster City, CA). The chromatography was performed using a 100 mm × 2.1 mm id 2.6 μm Kinetex Polar C18 100 Å (Phenomenex, Macclesfield, UK) with a flow rate of 500 μL/min and an injection volume of 5 μL. The temperatures of the column oven and autosampler were set at 30 and 4 °C, respectively. Two mobile phases were used in a linear gradient system: A: 92% water, 4% MeOH, 4% isopropanol and B: 92% acetonitrile, 4% MeOH, 4% isopropanol. Each mobile phase contained 20 mM of acetic acid. The LC method started with 0% mobile phase B 0–1 min, followed by a 1.6-min gradient to 45.6% B. Subsequently, B was further increased to 83.7% within 0.2 min and maintained at 83.7% for 0.7 min. The gradient was finally returned to the initial condition within 0.2 min, and the column was re-equilibrated with the initial gradient condition for 3.3 min before the next injection. All other LC–MS/MS parameters were as previously described [38]. For confirmation of the presence of the compounds, control of quantifier/qualifier MS-transition ratios were applied. Analyst Software (version 1.6.2) was used for instrument control, data acquisition, and subsequent quantifications. Quantifications were done on the basis of standard curves prepared in the range 0.0485 to 25 ng ml^−1^. Data points of the standard curves were weighted according to *x*^−1^.

### Sequence data analysis

Analysis of sequence reads was performed in QIIME version 1.9 [39]. The paired-end raw reads sorted out based on indices were joined using version 2.6 [40] with an overlapping minimum read length of 30 base pairs while removing reads with quality Phred scores less than 30 and other default parameters. In addition, the internal barcode, forward and reverse primers, and reads less than 200 base pairs were removed using the command split_libraries.py. Sequences were dereplicated, screened for chimeras, and clustered using vsearch version 2.6 [40]. For the fungal reads, ITS extraction was carried out before the clustering step using ITSx extractor version 1.0.6 [41]. Taxonomy assignments for the clustered operational taxonomic units (OTUs) were done using the GreenGenes 16S rRNA gene reference database for bacteria [42] and the UNITE database version 7.2 for fungal OTUs [43] in QIIME using assign_taxonomy.py. OTUs unassigned at kingdom level or assigned as chloroplast or mitochondrial sequences were removed from the OTU table. In addition, OTUs represented in < 3 samples in the total dataset were excluded.

### Statistical analysis

All statistical analyses and data visualizations were carried out in R v3.4.4 [44]. OTU tables, mapping files, and a tree file (in case of bacteria) were exported in R. Diversity analysis, species richness, and community dissimilarity analysis were carried out using the “vegan” package [45] and phyloseq [46]. For estimating alpha diversity, samples containing less than 2000 reads for bacteria and 500 for fungal were removed, and the OTU table was thus rarified 100 times at a depth of 2000 reads for bacteria and 500 reads for fungi and the mean of the diversity estimates of 100 trials was used. Observed OTU richness and Shannon diversity measures were used to estimate alpha diversity, and the significance of differences between alpha diversity was evaluated using Kruskal-Wallis rank sum test. For beta diversity-based calculations, samples representing less than 2000 reads were excluded and the OTU table was transformed to relative abundance. For beta diversity and partitioning of variance, UniFrac-weighted matrices for bacteria communities and Bray-Curtis for fungal communities were subjected to permutational analysis of variance (PERMANOVA) using the “adonis” test from the “vegan” package. Dissimilarity matrices for bacterial and fungal datasets were visualized using unconstrained principal coordinates analysis (PCoA). Sub-setting of the whole dataset based on compartment (root, rhizosphere, bulk soil, and shoot) was carried out in order to identify major variance components within compartments. In addition, data was split for each genotype within compartment in order to partition the variance. Indicator species associated to each mutant line [47] were identified. Highly significant OTUs (*p* < 0.01) with an indicator value of at least 0.4 were used to define indicator species [47].

### Correlation and network analysis

Spearman’s rank correlations of BX content in roots and shoots against each fungal and bacterial OTU were carried out. All the root and shoot samples at 10 DAS were used. For root samples, both fungal and bacterial OTUs were subjected to correlation analysis while we only had data for fungal OTUs from shoots. We only considered the BX-OTU correlations where OTU read numbers and BX contents were > 0 for at least eight samples (30% of total samples). Correlations with Spearman’s rho ≥ 0.5 and ≤ − 0.5 with *p* value < 0.01 were considered as significantly correlated. For the root microbial co-occurrence network construction, root data was split, and only W22_1 and its mutant samples were used. Bacterial and fungal OTUs were pooled, subjected to trimmed means of M transformation, and normalized as relative abundance counts per million using the “edgeR” package. We used OTUs that were present in at least 10 samples and which had Spearman’s rank correlations > 0.6 for positive correlations and < − 0.6 for negative correlations, and *p* values < 0.001. All correlated OTUs were visualized in a network, where OTUs were set as nodes, and the correlation as edges. OTUs that were identified as indicator OTUs in an indicator analysis and that also appeared in the co-occurrence were shown as bigger nodes. All the correlations were visualized using networks, and network properties were computed using the “igraph” package. All networks were subjected to Fruchterman-Reingold layout with 999 permutations. Description of specific analyses and R packages are described in the supplementary information (Additional file 2).

## Results

### Benzoxazinoids vary across mutants and their genetic background

**Fig. 1:**
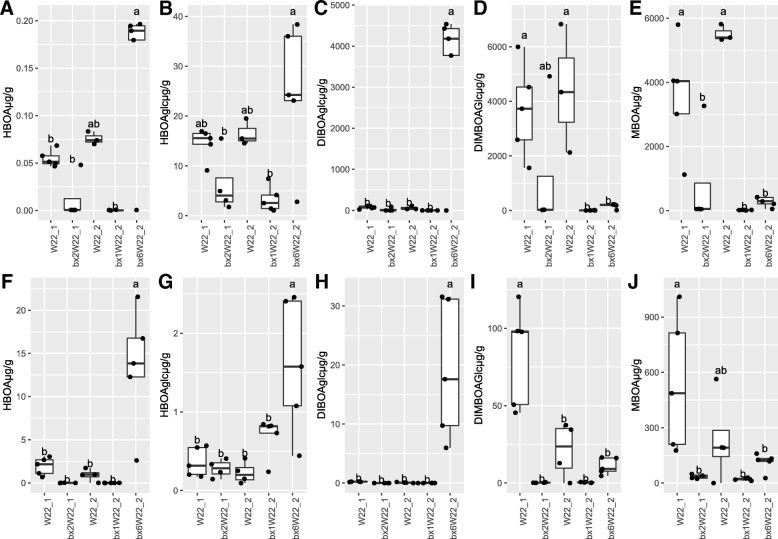
Quantities of BXs detected in the maize tissues of parental lines and mutants. The content of BXs in shoot tissues are shown in the upper row (**A**, **B**, **C**, **D**, **E**) and in root tissues in the lower row (**F**, **G**, **H**, **I**, **J**). Multiple comparison of treatments was carried out using HSD test and lowercase letters represent significant difference between genotypes

BXs were quantified at 10 DAS (Additional file 1: Table S1). Significant differences in BX profiles were observed between the mutants and their genetic background parental lines. Generally, BX accumulation was much higher in shoots compared to roots (Fig. 1). In both roots and shoots, *bx1*W22_2 and *bx2*W22_1 had lower amounts of BXs than *bx6*W22_2 and the parental lines. The Bx1 and Bx2 genes respectively code for enzymes upstream in the BX pathway for conversion of indole-3-glycerolphosphate to indole and indole to indolin-2-one, whereas Bx6 codes for downstream enzyme converting DIBOA-Glc to TRIBOA-Glc [48] (Additional file 1: Figure S2). HBOA-Glc was, however, found in relatively high amounts in the roots of *bx1*W22_2. As expected, amounts of DIBOA-Glc, BOA, HBOA, and HBOA-Glc that are produced upstream of the Bx6 gene were high in roots and shoots of *bx6*W22_2 (Fig. 1 and Additional file 1: Figure S3). Conversely, downstream products such as DIMBOA-Glc and its derivatives MBOA, HMBOA, and HMBOA-Glc were lower in bx6W22_2 in both roots and shoots compared to the parental line (Fig. 1, Additional file 1: Figure S3). For unknown reasons, DIMBOA-Glc and MBOA in roots were higher in W22_1 than in W22_2 parental lines.

In addition, we quantified BXs in the rhizosphere of W22_1 and its mutant bx2W22_1 at different DAS. We only detected MBOA and HMBOA. The results indicated higher BX amounts in the parental lines with almost negligible levels in the mutant lines (Additional file 1: Table S2).

### Sequence data

Reads from the shoot samples for the bacterial community analysis were heavily contaminated by plant chloroplast and mitochondrial reads and were thus removed from the dataset. After quality control, chimera filtering, and removal of chloroplast and mitochondrial reads, we obtained 2,850,289 reads of bacterial origin that were clustered into 8115 OTUs from 341 samples (out of 350). For the fungal library, we obtained 8,920,527 reads from 450 samples after quality control and removal of non-fungal reads; these reads were clustered into 763 OTUs. The numbers of reads across each compartment and sampling point for both bacterial and fungal dataset are shown in Additional file 1: Figure S4. In addition, the median averages and ranges of reads for both bacteria and fungi in each plant compartment are provided (Additional file 1: Table S3).

### Distinct microbial communities across shoots, bulk soil, rhizosphere, and root

Our analyses revealed distinct bacterial and fungal communities in the roots in comparison with communities in the rhizosphere and bulk soil. Root microbial communities had a higher proportion of *Proteobacteria* and *Sordariomycetes* than rhizosphere and bulk soil communities (Additional file 1: Figures S5A-S6A). Alpha diversity (observed OTU richness and Shannon diversity) was significantly lower in roots than in rhizosphere and bulk soil communities for both bacterial (*P* < 0.001) and fungal (*P* < 0.001) communities and was lowest in shoots in the case of fungal communities (*P* < 0.001) (Additional file 1: Figures S5B, S6B, and Table S4). A distinct bacterial beta diversity was revealed across roots, rhizosphere, and bulk soil (adonis, *R*^2^ = 0.46, *P* < 0.001) (Table 2, Additional file 1: Figure S5C). Likewise, plant compartment showed distinct clustering and explained a large part of the variation in fungal communities (adonis, *R*^2^ = 0.40, *P* < 0.001) in which shoot samples were also included (Table 2, Additional file 1: Figure S6C). Reduced alpha diversity in fungal communities in the shoots was observed, which were highly enriched with *Dothideomycetes* (Additional file 1: Figure S7).

**Table 2:** Permutation analysis of variance (PERMANOVA) using “adonis” test on UniFrac-weighted matrices for bacterial and Bray-Curtis distance matrices for fungal community dissimilarity assessment using 1000 permutations

Data set	Factor	Bacteria (*R*^2^)	Fungal (*R*^2^)
Whole	Compartment	0.46***	0.40***
Root	Genotype	0.17**	0.13***
	DAS	0.16***	0.04***
	Genotype*DAS	0.13**	0.11***
Rhizosphere	Genotype	0.13***	0.14***
	DAS	0.02*	0.03***
	Genotype*DAS	0.21***	0.11***
Rhizosphere_W22_1	Genotype	--	0.08***
	DAS	--	0.07*
	Genotype*DAS	0.24**	0.05*
Rhizosphere_W22_2	Genotype	0.18***	0.13***
	DAS	--	0.05***
	Genotype*DAS	0.18***	0.12***
Root_ W22_1	Genotype	0.20**	0.13***
	DAS	0.21***	0.06*
	Genotype*DAS	--	0.07**
Root_ W22_2	Genotype	0.13**	0.11***
	DAS	0.14***	0.07***
	Gen*DAS	0.19***	0.10***

Significance of test indicated as *** for *p* < 0.001, ** *p* > 0.01, *p < 0.05, and *R*^2^ for proportion of variation explained. Root and rhizosphere microbial dataset split based on genetic background of W22_1 and W22_2

### Distinct composition of rhizosphere, root, and shoot microbiomes in BX mutants and their background parental lines

Considering that the compartment had a major effect on the structure of both bacterial and fungal communities, we split the dataset and analyzed the rhizosphere, root, and shoot samples separately. We determined the effect of BXs on maize-associated microbiomes by comparing community data obtained from BX knock-out mutants and control parental lines.

**Fig. 2:**
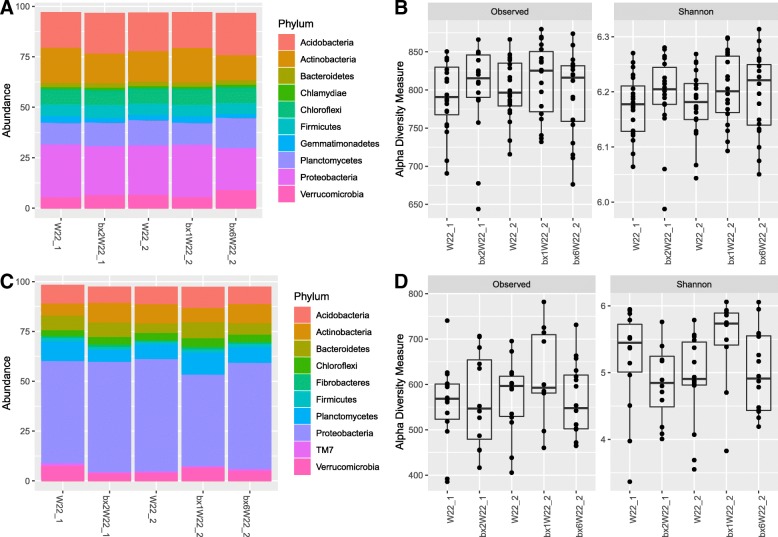
Taxonomic profiles and alpha diversity of bacterial communities across maize genotypes in rhizosphere and roots. Relative abundances of the top 10 phyla are shown in the rhizosphere (**a**) and root (**c**). The alpha diversity was estimated using observed OTU richness and Shannon diversity index for rhizosphere (**b**) and root (**d**) compartments

**Fig. 3:**
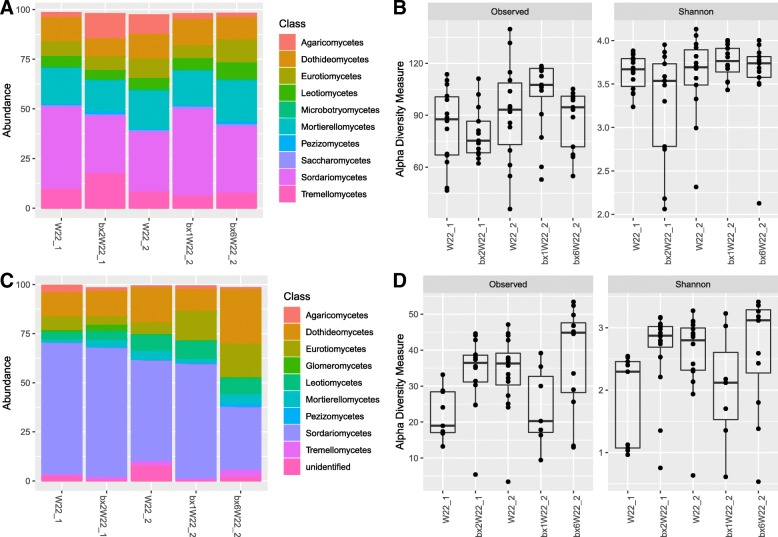
Taxonomic profile and alpha diversity of fungal community across maize genotypes in rhizosphere and roots. Relative abundance of top 10 classes is shown in the rhizosphere (**a**) and root (**c**). The alpha diversity was estimated using observed OTU richness and Shannon diversity index for rhizosphere (**b**) and root (**d**) compartments.

The difference in relative abundance of bacteria at phylum level and fungi at class level between parental lines and their respective mutants was minor both in the roots and in the rhizosphere (Figs. 2a, c and 3a, c). Likewise, differences in alpha diversity across parental lines and their mutants were not statistically significant in the rhizosphere or in the roots (Fig. 2b, d) except in root fungal communities of W22_1 and its mutant (Fig. 3b, d). In the roots, the *bx2*W22_1 mutant had significantly higher observed OTU richness (*P* < 0.007) and Shannon diversity index (*P* < 0.003) than its background control W22_1. On the contrary, fungal alpha diversities did not differ between W22_2 and its mutants (*bx1*W22_2 and *bx6*W22_2) (Fig. 3d, Additional file 1: Table S5). We found no differences in shoot fungal alpha diversities between the different maize lines (Additional file 1: Figure S7). Comparing shoot fungal abundances in W22_2 and its mutant lines, *Dothideomycetes* and *Sordariomycetes* were enriched in *bx6*W22_2 (Additional file 1: Figure S7).

A beta diversity-based analysis showed that a large proportion of the variation in the community structure of root and rhizosphere bacterial and fungal communities could be explained by genotype (parental lines and their mutants), DAS, and their interaction (DAS × genotype) (Table 2). Similarly, these factors also significantly shaped shoot fungal communities (Additional file 1: Table S6). Since the mutants used in this study had two different genetic backgrounds, the rhizosphere and root datasets were further split into the W22_1 and W22_2 parental lines and their respective mutants. PERMANOVA analysis revealed a higher genotype effect in roots in both bacterial (*R*^2^ = 0.20, *P* > 0.01) and fungal datasets (*R*^2^ = 0.13, *P* < 0.001) in W22_1 compared to W22_2 bacterial (*R*^2^ = 0.13, *P* > 0.01) and fungal communities (*R*^2^ = 0.11, *P* < 0.001). A genotype effect in the rhizosphere was only observed in W22_2 for the bacterial communities but in both W22_1 and W22_2 for the fungal communities. DAS explained a large variation in root bacterial communities, and in contrast, no effect was seen in rhizosphere bacterial communities for both genotypes. The DAS effect on fungal communities was comparable for both root and rhizosphere for W22_1 and W22_2. Genotype × DAS variance was highest in bacterial rhizosphere communities in both genotypes (Table 2).

**Fig. 4:**
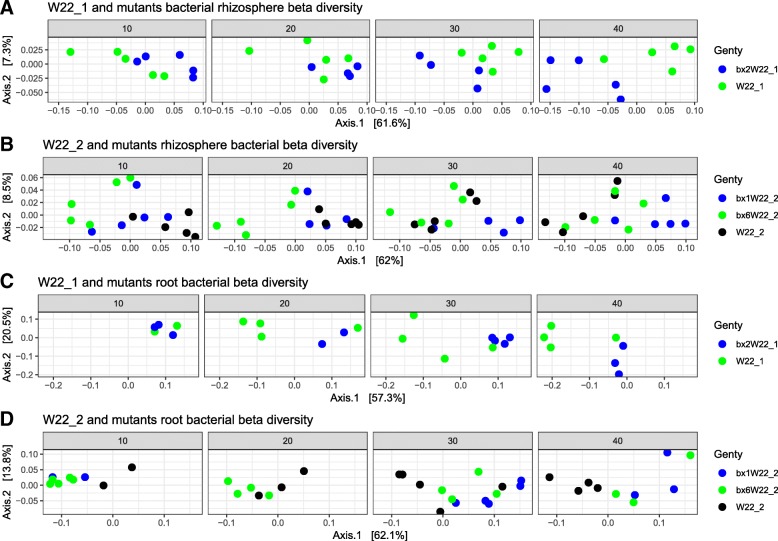
PCoA of bacterial communities using weighted UniFrac for the maize rhizosphere and roots. Parental lines and their corresponding mutants are shown separately in the rhizosphere (**a**, **b**) and roots (**c**, **d**). Samples are colored for each genotype. W22_1 and its mutants (**a**, **c**) and W22_2 and their mutants (**b**, **d**) are shown for each DAS

**Fig. 5:**
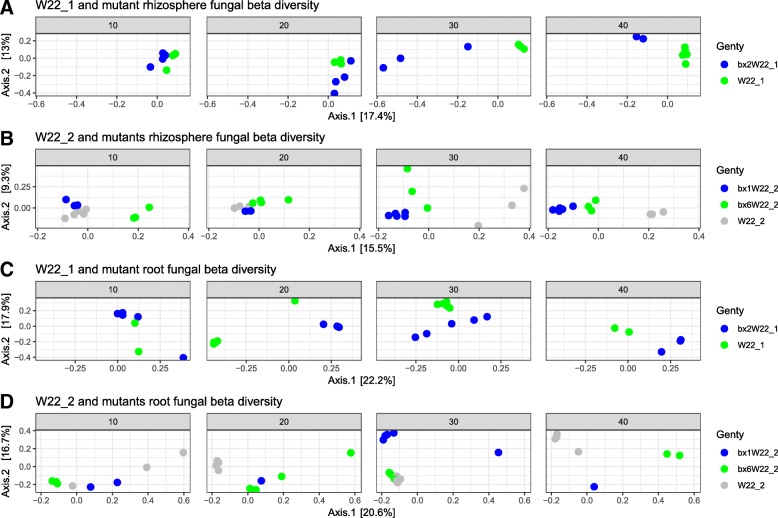
PCoA of fungal community using Bray-Curtis distances for the maize rhizosphere and roots. Parental lines and their corresponding mutants are shown separately in the rhizosphere (**a**, **b**) and roots (**c**, **d**). Samples are colored for each genotype. W22_1 and its mutants (a, c) and W22_2 and their mutants (b, d) are shown for each DAS

The visualization of distance matrices using PCoA analysis showed clustering in W22_1 and W22_2 according to genotype for both bacterial (Fig. 4) and fungal communities (Fig. 5) in the root and rhizosphere compartments for each individual DAS. However, clustering was hardly visible in shoot fungal communities (Additional file 1: Figure S8). As expected, clustering of bacterial and fungal communities in the root and rhizosphere was generally more pronounced at 30 and 40 DAS in comparison with the early sampling times (10 and 20 DAS) (Figs. 4 and 5). We detected bacterial taxa that were significantly different (mostly enriched) with increasing DAS (Additional file 1: Table S7). For specific days of sampling, genotype explained a large part of the variation in beta diversities of both fungal and bacterial communities after splitting data into each DAS (Additional file 1: Table S8). The genotype effect varied based on DAS and plant compartments, and for some DAS, where we only had few samples, the effect was not significant (Figs. 4 and 5).

### Correlation analysis of microbial communities and BX content

**Fig. 6:**
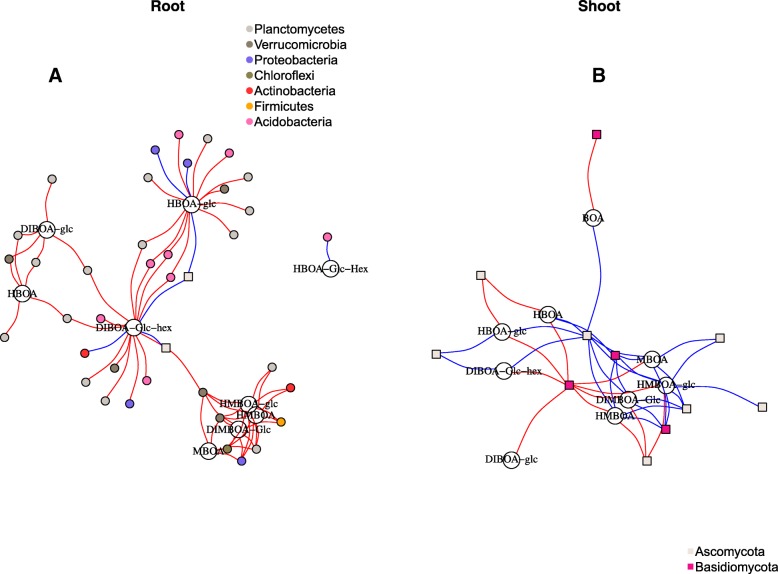
Network showing bacterial and fungal OTUs correlated to different BX metabolites. BX-OTU correlations in **a** roots and in **b** shoots at 10 DAS. OTUs and BX metabolites are shown as nodes with different size, and the correlation is shown as edges in the network. Positive and negative correlations are shown with blue and red edges, respectively. Bacterial and fungal nodes are represented as squares and circles, respectively

In order to identify OTUs correlating with BX content, we correlated the generated OTU table with concentrations of BX compounds from samples at 10 DAS (Additional file 1: Tables S7–S8). The BX metabolites with their correlating OTUs were visualized in a network for the root (Fig. 6a) and shoot datasets (Fig. 6b). In both bacterial and fungal communities, OTUs correlated positively as well as negatively with several BX metabolites (Additional file 1: Tables S9–S10). In the root dataset, the metabolites DIBOA-Glc-hex and HBOA-Glc showed correlation with the highest number of OTUs (Additional file 1: Table S11). Generally, we identified mostly negative correlations, whereas positively correlated OTUs were found in strikingly few bacterial and fungal taxa. Most OTUs within the bacterial phyla *Acidobacteria*, *Verrucomicrobia*, *Planctomycetes*, and *Chloroflexi*, correlated negatively with one or several BX compounds in the roots. On the contrary, within the phylum *Proteobacteria*, some members of *Alphaproteobacteria* interacted negatively while other members of *Betaproteobacteria* interacted positively with BXs (Additional file 1: Table S9). For the fungal root dataset, only a few OTUs correlated with BXs compared to the bacterial dataset, and these were all positive. Members of the families *Pleosporaceae* and *Pyronemataceae* correlated positively with DIMBOA-Glc and HBOA-Glc (Additional file 1: Table S9). In the shoots, HMBOA-Glc, MBOA, HMBOA, and DIMBOA-Glc showed correlation with the highest number of fungal OTUs, whereas HBOA-Glc-Hex, DIBOA-Glc, and BOA correlated with a relatively low number of OTUs (Additional file 1: Table S10). Fungal genera such as *Stemphylium*, *Vishniacozyma*, and *Didymella* correlated positivity with different BXs, whereas *Filobasidium*, *Blumeria*, *Ramularia*, and *Puccinia* correlated negatively with several BX compounds (Additional file 1: Table S10).

As a complement to the correlation analysis, indicator species analysis was carried out in order to identify OTUs which were significantly different in the parental lines or BX mutants in the root datasets for both the bacterial and fungal communities. Unlike the aforementioned correlation analysis which was carried out only at 10 DAS, indicator species analysis was carried out for the whole root dataset covering the four sampling times (10, 20, 30, 40 DAS). The W22_1 and W22_2 and mutants datasets from roots were analyzed separately. In the bacterial as well as fungal datasets, a large number of OTUs were found to be indicators for *bx2*W22_1 (Additional file 1: Tables S12–S15). Members of the bacterial phyla *Gemmatimonadetes*, *Chloroflexi*, *Actinobacteria*, and *Planctomycetes* (only order Gemmatales) were enriched in bx2W22_1 (Additional file 1: Table S12). Fungal genera such as *Gibellulopsis*, *Acremonium*, *Humicola*, *Sarocladium*, and several others were present in significantly higher amounts in the *bx2*W22_1 (Additional file 1: Table S14). The fungal genus *Stemphylium* and bacterial OTUs within *Verrucomicrobia*, *Planctomycetes* (mostly order Pirellulales and WD2101), and *Acidobacteria* were identified as indicator species for the wild type W22_1 (Additional file 1: Table S12, S14). Similar to our results in *bx2*W22_1, a large number of bacterial OTUs were identified as indicator species for *bx1*W22_2 as compared to W22_2 and *bx6*W22_2 (Additional file 1: Table S13, S15). Bacterial OTUs assigned to the phyla *Chloroflexi*, *Acidobacteria*, *Bacteroidetes*, *Planctomycetes*, and *Proteobacteria*, among others, were enriched in *bx1*W22_2. W22_2 showed enrichment of a few members of *Proteobacteria* and *Actinobacteria*, and in *bx6W22_2*, members of *Actinobacteria* were enriched (Additional file 1: Table S13). Surprisingly, no fungal indicator species were detected for *bx1*W22_2; however, few OTUs were indicator species for W22_2 mostly belonging to *Sordariomycetes*. Only four fungal OTUs were identified as indicator species in *bx6W22_2* (Additional file 1: Table S15).

### Co-occurrence analysis of root communities in W22_1 and its mutant

**Fig. 7:**
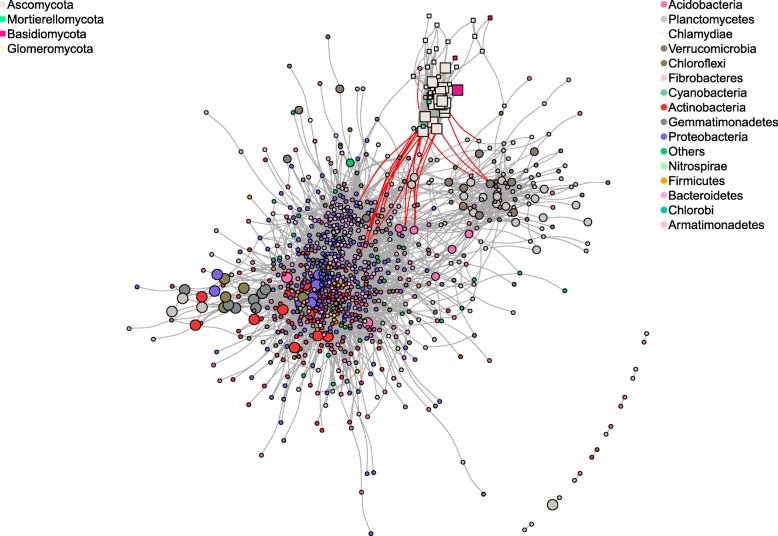
Microbial network based on Spearman’s correlations in the roots of W22_1 and its mutant. OTUs are shown as nodes, and correlations as edges. Positive and negative correlations are shown with grey and red edges, respectively. Bacterial and fungal nodes are represented as square and circle symbols in the network, respectively. Indicator OTUs for *bx1*W22_1 and W22_1 are shown with a large and a medium node size while others are shown with small size to indicate the location of indicator species in overall network

In order to clarify whether the identified indicator OTUs for BX were major players in the overall root OTU network, we performed a co-occurrence network analysis including root microbial communities of W22_1 and its mutant. Both positive and negative correlations were identified in the bacterial and fungal communities. The correlations were mostly positive (10051) with only a few negative (20) among 1014 OTUs as visualized in the network (Fig. 7). Positive correlation was mostly found within bacteria (9554) or within fungi (495), whereas negative correlations were mostly observed between fungi and bacteria (19) (Additional file 1: Table S16). There were three main clusters of indicator OTUs identified, one comprising fungal OTUs and the other two bacterial OTUs (Fig. 7). We identified negative correlations between fungal and bacterial OTUs, and surprisingly, most of the fungal OTUs (7 out of 9 OTUs) were indicator species of bx2W22_1 (Additional file 1: Table S16). Highly connected hub OTUs were identified as the top five percent of the OTUs having the most correlations in the network. Only six bacterial OTUs out of the 48 hub OTUs were also indicator species either for parental or mutant line (Additional file 1: Table S16).

## Discussion

The effect of plant genotype on plant-associated microbial communities is well documented [49–54]. Despite the univocal evidence of plant genotype effects on microbiome assemblage, and that a repertoire of plant exudates including secondary metabolites unique for each plant genotype drives microbial assemblage, little research attention is directed towards the mechanisms involved. In this study, we used well-characterized BX mutants and their respective parental lines to dissect the influence of the defensive secondary metabolite BXs on the maize microbiome.

### BX profiles in maize lines

Initially, we screened the maize lines to confirm their BX content in both roots and shoots at 10 DAS. Because the parent lines have the same genetic background (W22) and only differ in gene insertions (a1-m3::DS for W22_1 and r1:sc:m3 for W22_2) for anthocyanin, we speculated that the lower BX levels detected in the W22_2 parental line could be caused by gene interactive effects arising from the gene insertions [14, 55]. Hence, mutations that target anthocyanin (as is the case of the parental lines used in this study) are likely to alter BXs synthesis. Therefore, inferences of parental lines W22_1 and W22_2 and their effects on community composition were interpreted separately.

BX quantification showed significant reduction of several BX metabolites in mutants compared to the wild types. Differences in BX quantities between shoots and roots could be explained by transport mechanisms [14]. We expected a negligible amount of BXs in the *bx1*W22_2 mutant, primarily due to the mutation in the Bx1 gene located at the first step in the BX biosynthetic pathway and may entirely disrupt BX synthesis. However, small quantities were detected in the mutants, and this observation corroborates with earlier studies [56].

The detected BX residues in *bx1*W22_2 could be due to leakages originating from the two other pathways IGL1 (indole-3-glycerolphosphate lyase 1) and TSA1 (tryptophan synthase alpha-subunit 1). Similarly, for bx2W22_1 with minute BX levels, a complementary activity of Bx3, Bx4, or Bx5 genes or the catalytic activity of an unknown CYP71C protein may be responsible [14]. A notable accumulation of intermediate BX compounds were found in *bx6*W22_2 as also shown earlier [18]. This line has a mutation in the Bx6 gene located downstream in the BX pathway responsible for conversion of DIBOA-Glc to TRIBOA-Glc, the precursor for DIMBOA-Glc [13, 14, 57, 58]. This study also showed reduced amounts of DIMBOA-Glc and MBOA in *bx6*W22_2 roots corroborating the BX synthesis pathway.

Plant bioactive compounds present in root tissues are mostly released into the rhizosphere [59]. We confirmed BXs exudation into the rhizosphere by quantifying BXs in the rhizosphere of W22_1 and its mutant *bx2*W22_1. Our findings were consistent with BX levels in roots, where higher amounts were detected in W22_1 with extremely low to no detection levels in *bx2*W22_1.

### Microbial communities are structured by maize compartments

Compared to bulk soil or rhizosphere communities, our results confirmed previous findings that the roots harbor a lower diversity and distinct microbial communities. The root microbiome is reported to be influenced by plant genotype but also soil type which serve as source of inoculum [2, 5, 33]. The observed enrichment of *Proteobacteria* in maize roots is typical of plant-associated microbiomes [53, 60, 61] and is consistent with earlier studies on maize roots [62, 63]. Similarly, we observed distinct fungal communities across bulk soil, rhizosphere, and roots, and enrichment of a few fungal taxa as for instance *Sordariomycetes* in roots which was similar to earlier observations in *Arabidopsis thaliana* [64] and *Arabis alpine* [65], and could thus indicate a strong selection of this taxon by plant roots. *Sordariomycetes* are ubiquitous with ecosystem functions including decomposition and nutrient cycling and may be present as endophytes and pathogens of plants [66]. In the shoot compartment, the fungal class *Dothideomycetes* strongly dominated, corroborating with previous studies of phyllosphere microbial communities [67, 68]. The fungal diversity in the cereal phyllosphere is generally low compared to roots [50, 69] which could relate to limited nutrient resources in leaves and harsh environmental conditions such as high radiation and temperature fluctuations [70].

### BXs structure microbial communities

To investigate BXs, we assumed that belowground BX effect would be highest in the roots followed by the rhizosphere and with only minor effects in the bulk soil, and thus, roots and rhizosphere became the prime focus of the present study. We defined the root compartment as the root including the rhizoplane (the plant root surface), known to modulate the most dynamic microbial-root interactions [71]. We first tested BX effects on microbial alpha and beta diversities by comparing microbial communities in BX mutants and their parent lines. Despite the overall genetic similarity among the maize lines, we observed that BX mutations explained a large part of the variation in microbial community structures not only in roots (bacteria 17%; fungal 13%), but also in the rhizosphere (bacteria 13%; fungal 14%). After splitting the data into each parental line and its corresponding mutant(s), analysis of the most significant sources of variation revealed that W22_1 and its mutant contributed the largest variation in both bacterial (20%) and fungal (13%) communities in roots compared to a lower but significant effect observed in W22_2 and its mutants. These results indicate that genotypic effect correlated with levels of BX quantified; W22_1 with higher BXs levels exhibited higher genotypic effect while W22_2 with lower BXs had smaller effect on both bacterial and fungal communities. We therefore speculate that the degree of genotypic effect exerted by maize seedlings on the root microbiome is dependent on the level of BX present. Plant microbiomes are known to be altered by plant metabolites [71–74], and we corroborate these findings by presenting an in-depth role of specific secondary metabolites BXs as candidate exudates involved in assembling both below-and aboveground microbiomes of maize. Recent study also showed potential role of BX on maize root and rhizosphere-associated microbiota at late maturity stage and at one time point [75]. Our study however further expands the understanding of the mechanistic influence of BXs on the maize microbiome which is not limited to root or rhizosphere, but also in shoots, and in addition tracks BX-dependent effects at four different time points of early (40 days) plant development.

In addition to genotype effects, the DAS as well as genotype × DAS interaction explained a significant part of the variation in the microbial communities which may be attributed to plant traits that changes as the plant matures. Plant age for instance was reported as a major driver of microbial communities in the maize rhizosphere [76]. In order to eliminate DAS and genotype × DAS interaction effects, microbial communities were tested for genotype effects at each individual DAS. We consistently observed that the mutants and their parental lines explained a large part of the variation in the microbial community structure. Furthermore, PCoA plots split according to DAS showed clustering of the mutants and their parental lines. The clustering of the fungal and bacterial communities became more pronounced with increasing DAS both in the rhizosphere and in the roots. BX synthesis was previously reported to fluctuate in the first 6 weeks of growth, and further, there is a lag-phase between metabolite accumulation and effects on microbial communities [72, 77, 78].

Moreover, the effect of maize genotype on the rhizosphere bacterial and fungal communities was significant, and this confirms previous studies in which cereals were found to exert measurable influence on the rhizosphere [52, 62, 79]. Variation partitioning, however, revealed that genotypic effects were highly dependent on the maize line and there were strong genotype × DAS interaction effects on bacterial and fungal communities in the maize rhizosphere. An analysis of BX contents in the rhizosphere revealed BX levels lower than those detected in roots. Although only small amounts of BXs (MBOA and HMBOA) could be detected in the rhizosphere, quantities reflected those found in roots of W22_1 and its mutant.

### Gatekeeper effect of BXs

To evaluate whether BX metabolites allow selective enrichment of members of the microbial communities in roots, we compared communities of W22_1 (parental line with highest amount of BXs) and its mutant. W22_1 showed significantly lower fungal alpha diversity indicating that antagonistic properties of BXs could form part of the mechanisms that characterize the gatekeeper role at the rhizoplane, thus preventing a range of microbial species from entering into the root compartment. Consistent with the proposed multistep selection model for root microbiota differentiation [79], genotypic traits including plant metabolites strongly influence microbial selection in the root (including the rhizoplane) compartment [71]. In the present study, however, the reduced fungal diversity in roots of the parental lines was not observed in bacterial communities, implying that BXs could have a higher impact on fungal communities.

An analysis carried out for parental lines and their mutants confirmed that a large number of indicator species could be identified in the mutants for both bacterial and fungal communities, with the highest number in *bx2*W22_1, including species such as *Actinobacteria*, *Chloroflexi*, and *Gemmatimonadetes*, phyla that were almost absent in the wild-type W22_1. On the contrary, significant enrichment of members of the phyla *Verrucomicrobia* and *Acidobacteria* was observed in the W22_1 parental line. The fungal genera *Gibellulopsis*, *Acremonium*, *Humicola*, and *Sarocladium* were identified as indicator species in bx2W22_1, whereas only *Stemphylium* was found to be enriched in the parental line. These results support the gatekeeper effect of BXs that are preventing a wide range of microorganisms from entering the root compartment.

### BX quantities correlate with specific OTUs

To specifically target the effects of BX compounds on microbial communities, we performed correlation analysis of BX concentrations in plants against the relative abundance of OTUs at 10 DAS. Both positive and negative correlations between fungal and bacterial OTUs and BX metabolites in roots and shoots were identified. Interestingly, positive correlations among bacterial OTUs were mostly observed among members of the *Proteobacteria*, and negative correlations consisted of OTUs within *Acidobacteria*, *Verrucomicrobia*, *Planctomycetes*, and *Chloroflexi*. Only a few fungal taxa among *Ascomycota* correlated with BX contents compared to bacterial taxa, which could be explained by the fact that active recruitment of bacteria could happen earlier than fungi, or it could simply be caused by the large diversity of bacteria in the roots in comparison with fungi. In the shoots, fungal genera such as the pathogens *Blumeria*, *Ramularia*, and *Puccinia* along with the yeast *Filobasidium* were negatively correlated to BX contents. Because these pathogens were deterred by BXs already at an early growth stage (10 DAS), we speculate that BXs could protect against foliar pathogens. Several studies have similarly reported BX fungicidal effects on cereal pathogens. For example, the fungi *Helminthosporium turcicum*, *Cephalosporium maydis*, *Fusarium moniliforme*, *Fusarium subglutinans*, *Fusarium culmorum*, *Gaeumannomyces graminis*, *Microdochium nivale*, and *Puccinia graminis* were found to be negatively correlated to BX content [80–91].

Besides the antagonistic effects of BXs, we found that some fungal genera including *Stemphylium*, *Vishniacozyma*, and *Didymella* positively correlated with BX content in shoots. Similar to our findings, root exudates of wheat, which like maize contains high DIMBOA quantities, affected rhizosphere fungal communities [92]. In this work, Xu et al. [92] detected enrichment of the fungal class *Sordariomycetes* and the bacteria taxa *Alphaproteobacteria*, *Actinobacteria*, and *Gemmatimonadetes*, while others such as *Gammaproteobacteria*, *Sphingobacteria*, *Cytophagia* and fungal taxa *Pezizomycetes* and *Eurotiomycetes* were depleted in a watermelon-wheat rhizosphere. It was further shown that these shifts in the rhizosphere microbiome coincided with a significant decline in *Fusarium oxysporium* f.sp*.niveum* in the watermelon-wheat system [91]. Altogether, BXs evidently have selective effects on cereal-associated microbial communities. However, results of the BX-correlated taxa may vary depending on the soil inoculum.

### Co-occurrence and microbial networks

In order to dissect whether microbial communities in the roots of maize lines were modulated by microbe-microbe interactions, we performed Spearman correlations. By doing this, we observed both positive and negative microbial interactions. Co-occurrence analysis of the microbial communities in W22_1 roots showed distinct network patterns. Although the correlation analysis hardly explains cause and effect, it reveals highly connected OTUs, regardless of their relative abundance. The most highly connected OTUs could potentially act as keystone OTUs that could be considered as major players in the formation of microbial communities [93]. We defined highly connected OTUs as the top five percent OTUs with the highest number of connections and checked whether there was any overlap with OTUs affected by BX content (indicator OTUs). Notably, only six bacterial indicator OTUs were defined as highly connected, thus demonstrating that BX effects were not on the core network. BX indicator species could be found in three distinct co-occurrence clusters in the periphery of the microbial network suggesting that BXs are targeting specific groups of taxa. Surprisingly, most of the negative correlations were observed between bacterial and fungal OTUs. Earlier studies also proposed similar negative correlations between bacteria and fungi suggesting specific inter-kingdom interactions in the roots and rhizosphere [64]. This could be caused by bacterial-fungal competition for scarce resources [94] or via production of antimicrobial compounds [95]. We further observed that fungal OTUs involved in fungal-bacterial negative interactions were mostly indicators of BX mutants suggesting that BX could be a vital part of the microbial inter-kingdom warfare, and thus, BXs could be targeting specific members of the microbiome.

## Conclusions

As a step forward towards understanding how the plant structures its associated microbiome, we have shown that BXs plays a key role and that these compounds are vital for the fine-tuning of the microbiome in maize. By using maize knock-out mutants impaired in BX genes, the present study demonstrated the role of BXs in microbial community assemblage for not only bacterial but also for fungal communities during the early stages of maize development. Using indicator species and network analysis, we showed that BXs affected only a subset of the microbiota. The example of negative correlation of BX against fungal pathogens could indicate a potential for BXs in biological control of pathogens. A higher BX synthesis during the early development of maize and its effect on microbial communities might open new frontiers for maize breeding in future.

## Additional files

Additional file 1: Supplementary figures and tables. This file contains supplementary Figures S1–S8 and Tables S1–S16. (ZIP 1563 kb)

Additional file 2: Supplementary files (SFL). The supplementary file consist of all the raw files (such as OTU tables, mapping files) and data analysis used in this study. (ZIP 331 kb)
