# Supplementary material for: Maize synthesized benzoxazinoids affect the host associated microbiome
Source: Microbiome. 2019 Apr 11;7:59. doi: 10.1186/s40168-019-0677-7 (PMC6460791; doi:10.1186/s40168-019-0677-7)
Supplement: Supplementary file 1 — Supplementary figures and tables. This file contains supplementary Figures S1–S8 and Tables S1–S16. (ZIP 1563 kb) [file 40168_2019_677_MOESM1_ESM.zip › Figure S1.pdf]

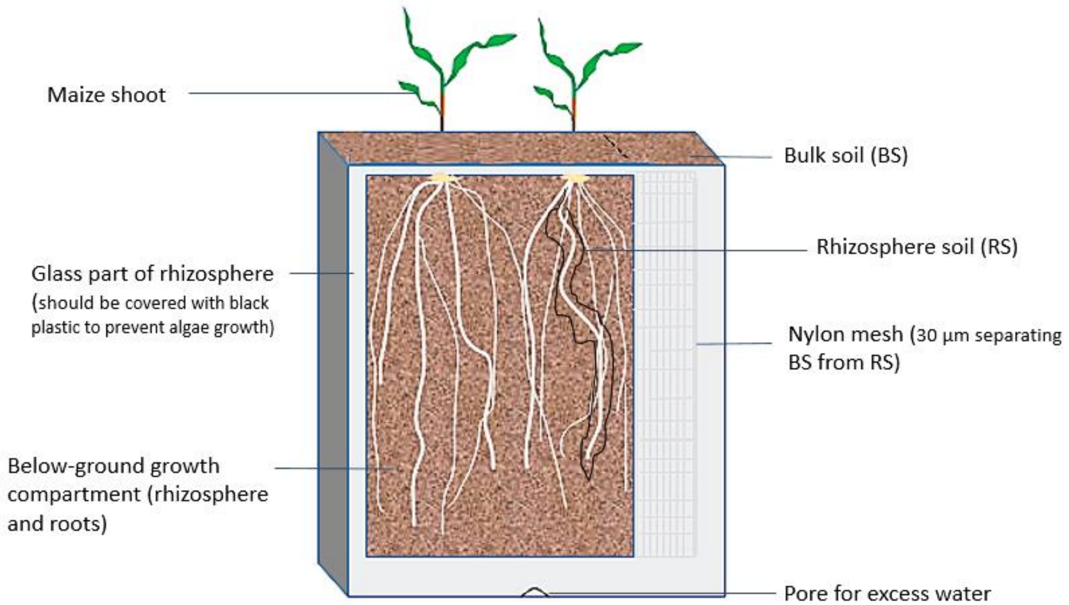

Fig. 1a Schematics of the rhizobox growth system. A cross-sectional view of the belowground growth compartment comprising maize roots and the rhizosphere is presented**.**

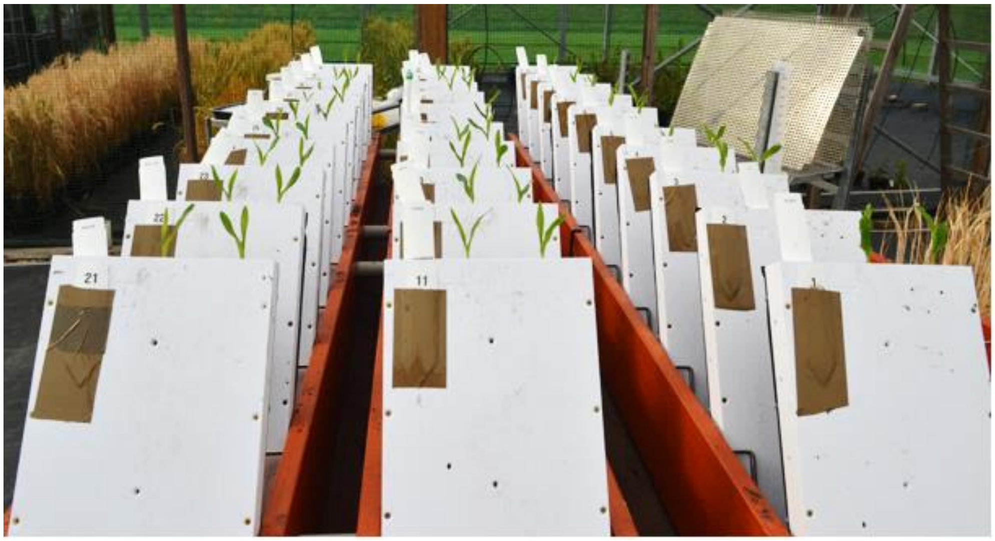

Fig. 1b Maize seedlings in rhizobox growth system.
