# Supplementary material for: Maize synthesized benzoxazinoids affect the host associated microbiome
Source: Microbiome. 2019 Apr 11;7:59. doi: 10.1186/s40168-019-0677-7 (PMC6460791; doi:10.1186/s40168-019-0677-7)
Supplement: Supplementary file 1 — Supplementary figures and tables. This file contains supplementary Figures S1–S8 and Tables S1–S16. (ZIP 1563 kb) [file 40168_2019_677_MOESM1_ESM.zip › Figure S3.pdf]

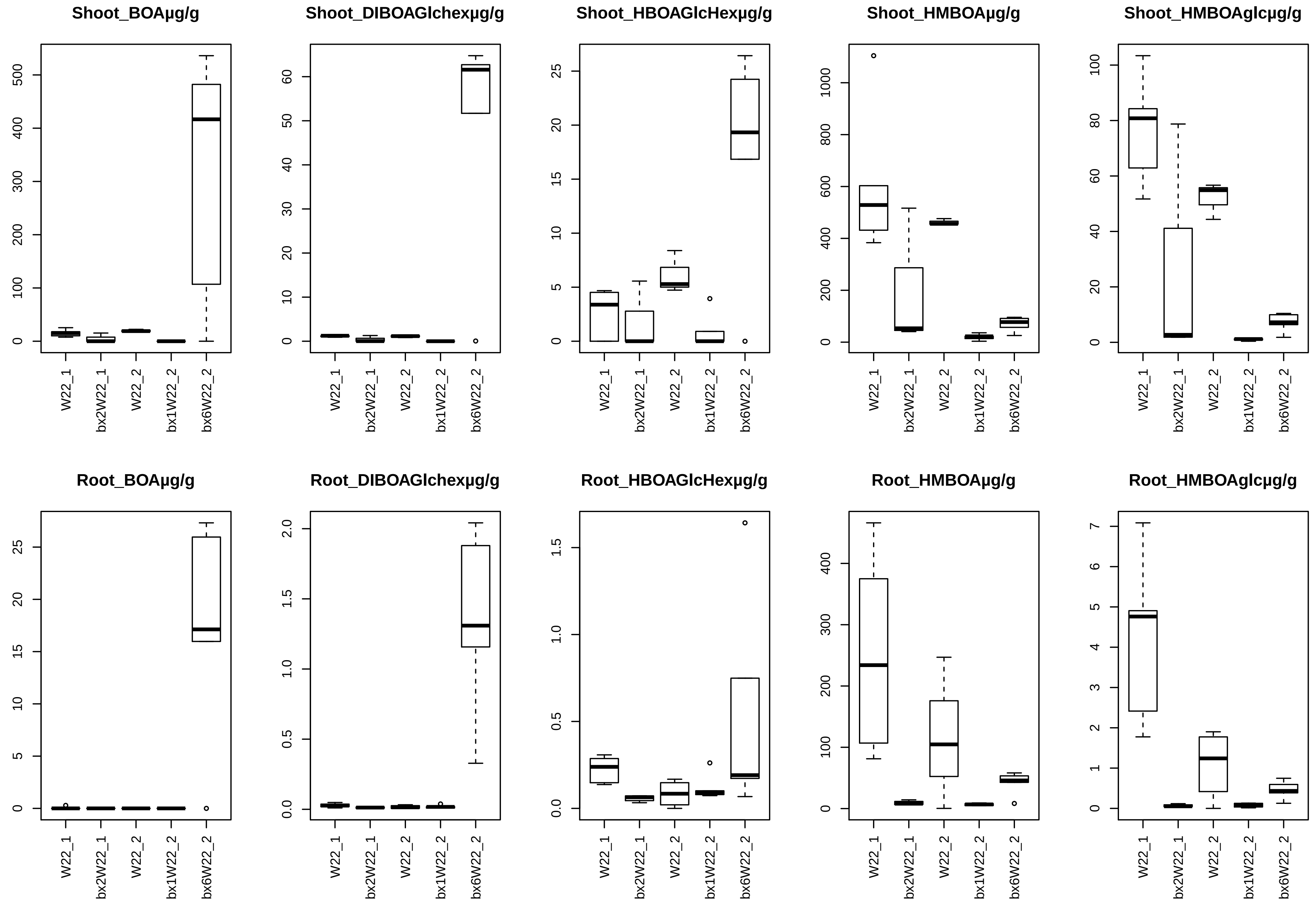
Shoot_BOAµg/g
Shoot_DIBOAGlchexµg/g
Shoot_HBOAGlcHexµg/g
Shoot_HMBOAµg/g
Shoot_HMBOAglcµg/g
Root_BOAµg/g
Root_DIBOAGlchexµg/g
Root_HBOAGlcHexµg/g
Root_HMBOAµg/g
Root_HMBOAglcµg/g
0
100
200
300
400
500
10
20
30
40
50
60
5
15
25
600
800
1000
80
0.0
0.5
1.0
1.5
2.0
1
2
3
4
6
7
W22_1
bx2W22_1
W22_2
bx1W22_2
bx6W22_2
