# Supplementary material for: Maize synthesized benzoxazinoids affect the host associated microbiome
Source: Microbiome. 2019 Apr 11;7:59. doi: 10.1186/s40168-019-0677-7 (PMC6460791; doi:10.1186/s40168-019-0677-7)
Supplement: Supplementary file 1 — Supplementary figures and tables. This file contains supplementary Figures S1–S8 and Tables S1–S16. (ZIP 1563 kb) [file 40168_2019_677_MOESM1_ESM.zip › S1-S6,S8_SupplementaryTables.docx]

Supplementary Table 1: Abbreviation and full name of different BX metabolites measured in this study from maize root, shoot and rhizosphere soil.

| **Benzoxazinoids** | **Acronym** |
| --- | --- |
| Benzoxazolin-2-one | BOA^1,2^ |
| 6-methoxy-2-benzoxazolinone | MBOA^1,2^ |
| 2-hydroxy-1, 4-benzoxazin-3-one | HBOA^1,2^ |
| 2-hydroxy-7-methoxy-1, 4-benzoxazin-3-one | HMBOA^1,2^ |
| 2, 4-dihydroxy-1, 4-benzoxazin-3-one | DIBOA^2^ |
| 2, 4-dihydroxy-7-methoxy-1, 4-benzoxazin-3-one | DIMBOA^2^ |
| 2-β-D-glucopyranosyloxy-4-hydroxy-1,4-benzoxazin-3-one | DIBOA-glc^1,2^ |
| 2-β-D-glucopyranosyloxy-1,4-benzoxazin-3-one | HBOA-glc^1,2^ |
| 2-β-D-glucopyranosyloxy-7-methoxy-1,4-benzoxazin-3-one | HMBOA-glc^1,2^ |
| 2-β-D-glucopyranosyloxy-4-hydroxy-7-methoxy-1,4-benzoxazin-3-one | DIMBOA-glc^1,2^ |
| 2-4- β-D-glucopyranosyloxy-4-hydroxy-7,8-dimethoxy-1,4-benzoxazin-3-one | DIM_2_BOA-glc^1^ |
| Double hexose derivative of 2,4-dihydroxy-1, 4-benzoxazin-3-one | DIBOA-glc-hex^1,2^ |
| Double hexose derivative of 2-hydroxy-1, 4-benzoxazin-3-one | HBOA-glc-hex^1,2^ |

Note: 1: Analyzed in root and shoot, 2: Analyzed in rhizosphere soil

Supplementary Table 2: MBOA and HMBOA quantification in rhizosphere soil of W22_1 and its mutant bx2W22_1. For quantification of Bx in soil, two different methods were used.

| SampleID | Genotype | MBOA1(ng/g ) | MBOA2(ng/g ) | HMBOA1(ng/g ) | HMBOA2(ng/g ) |
| --- | --- | --- | --- | --- | --- |
| R1BxRSB10 | bx2W22_1 | 0 | 0 | 0 | 0 |
| R2BxRSB10 | bx2W22_1 | 0 | 0 | 0 | 0 |
| R3BxRSB10 | bx2W22_1 | 0 | 0 | 0 | 0 |
| R4BxRSB10 | bx2W22_1 | 0 | 0 | 0 | 0 |
| R5BxRSB10 | bx2W22_1 | 0 | 0 | 0 | 0 |
| R1BxRSB20 | bx2W22_1 | 0 | 0 | 0 | 0 |
| R2BxRSB20 | bx2W22_1 | 0 | 0 | 0 | 0 |
| R3BxRSB20 | bx2W22_1 | 9.025766469 | 7.37041536 | 0 | 0 |
| R4BxRSB20 | bx2W22_1 | 0 | 0 | 0 | 0 |
| R5BxRSB20 | bx2W22_1 | 0 | 0 | 0 | 0 |
| R1BxRSB30 | bx2W22_1 | 0 | 0 | 0 | 0 |
| R2BxRSB30 | bx2W22_1 | 0 | 0 | 0 | 0 |
| R3BxRSB30 | bx2W22_1 | 0 | 0 | 0 | 0 |
| R4BxRSB30 | bx2W22_1 | 0 | 0 | 0 | 0 |
| R5BxRSB30 | bx2W22_1 | 0 | 0 | 0 | 0 |
| R1BxRSB40 | bx2W22_1 | 0 | 0 | 0 | 0 |
| R2BxRSB40 | bx2W22_1 | 0 | 0 | 0 | 0 |
| R3BxRSB40 | bx2W22_1 | 0 | 0 | 0 | 0 |
| R4BxRSB40 | bx2W22_1 | 0 | 0 | 0 | 0 |
| R5BxRSB40 | bx2W22_1 | 0 | 0 | 0 | 0 |
| R1BxRSD10 | W22_1 | 410.4696665 | 417.870708 | 81.88696217 | 84.2311555 |
| R2BxRSD10 | W22_1 | 0 | 0 | 0 | 0 |
| R3BxRSD10 | W22_1 | 140.3964002 | 140.7499701 |  |  |
| R4BxRSD10 | W22_1 | 0 | 0 | 27.03136516 | 23.13540431 |
| R5BxRSD10 | W22_1 | 222.4402636 | 222.4808474 | 38.0497846 | 38.77266662 |
| R1BxRSD20 | W22_1 | 46.24002112 | 45.11852394 | 0 | 0 |
| R2BxRSD20 | W22_1 | 84.09533624 | 86.49407166 | 14.60020345 | 11.7664813 |
| R3BxRSD20 | W22_1 | 181.6360201 | 177.47432 | 21.89794283 | 23.98387885 |
| R4BxRSD20 | W22_1 | 0 | 0 | 0 | 0 |
| R5BxRSD20 | W22_1 | 0 | 0 | 46.88410962 | 47.83606797 |
| R1BxRSD30 | W22_1 | 0 | 0 | 0 | 0 |
| R2BxRSD30 | W22_1 | 0 | 0 | 103.1310799 | 112.3153245 |
| R3BxRSD30 | W22_1 | 0 | 0 | 20.22088893 | 19.75692387 |
| R4BxRSD30 | W22_1 | 0 | 0 | 0 | 0 |
| R5BxRSD30 | W22_1 | 78.0290972 | 79.96882138 | 19.5321094 | 17.28817954 |
| R1BxRSD40 | W22_1 | 19.70696326 | 16.74751341 | 0 | 0 |
| R2BxRSD40 | W22_1 | 0 | 0 | 0 | 0 |
| R3BxRSD40 | W22_1 | 25.03927815 | 23.03143744 | 0 | 0 |
| R4BxRSD40 | W22_1 | 0 | 0 | 0 | 0 |
| R5BxRSD40 | W22_1 | 69.47167389 | 62.58744924 | 9.478429277 | 8.946876458 |

|  | Fungi | | | | Bacteria | | | |
| --- | --- | --- | --- | --- | --- | --- | --- | --- |
| Compartment | Mean | Median | Min | Max | Mean | Median | Min | Max |
| BS | 19399.65 | 1831 | 3 | 295092 | 9798.885 | 7650 | 2 | 38688 |
| RS | 24879.88 | 4003.5 | 48 | 255400 | 10117.31 | 7958.5 | 13 | 30011 |
| Rt | 6699.46 | 341.5 | 2 | 139942 | 4765.13 | 2988.5 | 25 | 17856 |
| SS | 28526.46 | 886 | 1 | 1044910 |  |  |  |  |

Supplementary Table 3: Mean, median and range of reads per compartment for fungal and bacterial libraries.

Supplementary Table 4: Alpha diversity in plant compartment pair wise comparison using Wilcoxon rank sum test.

| Community | Compartment | Observed | Shannon |
| --- | --- | --- | --- |
| Bacteria | BS-RS | ns | ns |
|  | BS-Rt | *** | *** |
|  | Rs_Rt | *** | *** |
| Fungal | BS-RS | ns | ns |
|  | BS-Rt | *** | *** |
|  | Rs_Rt | *** | *** |
|  | BS-SS | *** | *** |
|  | Rs-SS | *** | *** |
|  | Rt-SS | *** | ** |

Supplementary Table 5: Difference in genotype based on alpha diversity using Wilcoxon rank sum test for pair wise comparison.

| Community | Genotype | Observed | Shannon |
| --- | --- | --- | --- |
| Bacteria in root | W22_1 - bx2W22_1 | ns | ns |
|  | W22_2 – bx1W22_2 | ns | ns |
|  | W22_2 – bx6W22_2 | ns | ns |
|  | bx1W22_2- bx6W22_2 | ns | ns |
| Fungi in root | W22_1 - bx2W22_1 | ** | ** |
|  | W22_2 – bx1W22_2 | ns | ns |
|  | W22_2 – bx6W22_2 | ns | ns |
|  | bx1W22_2- bx6W22_2 | ns | ns |

Supplementary Table 6: Adonis test for partitioning of variance for genotype and days after sowing (DAS) in fungal shoot dataset.

| **Data set** | **Factor** | **Fungal (R^2^)** |
| --- | --- | --- |
| Shoot | Genotype | 0.09* |
|  | DAS | 0.10*** |
|  | Genotype*DAS | 0.10** |
| Shoot_W22_1 | Genotype | ns |
|  | DAS | 0.18** |
|  | Genotype*DAS | 0.09** |
| Shoot_W22_2 | Genotype | 0.10** |
|  | DAS | 0.07*** |
|  | Genotype*DAS | 0.09** |

Supplementary Table 8: Adonis test for rhizosphere, root and shoot dataset after splitting the W22_1 and W22_1 and their mutant genotype databased on days after sowing

| **Data set** | **Factor** | **Bacteria (R^2^)** | **Fungal (R^2^)** |
| --- | --- | --- | --- |
| Rhizosphere_W22_1_10 | Genotype | ns | ns |
| Rhizosphere_W22_1_20 | Genotype | ns | 0.29* |
| Rhizosphere_W22_1_30 | Genotype | 0.27* | ns |
| Rhizosphere_W22_1_40 | Genotype | 0.46* | 0.36* |
| Root_W22_1_10 | Genotype | ns | ns |
| Root_ W22_1_20 | Genotype | ns | 0.53* |
| Root_ W22_1_30 | Genotype | 0.35 (0.08) | 0.50* |
| Root_ W22_1_40 | Genotype | 0.50* | ns |
| Shoot_ W22_1_10 | Genotype | -- | 0.26* |
| Shoot_ W22_1_20 | Genotype | -- | ns |
| Shoot_ W22_1_30 | Genotype | -- | 0.67** |
| Shoot_ W22_1_40 | Genotype | -- | ns |
| Rhizosphere_B W22_2_10 | Genotype | 0.46* | 0.46*** |
| Rhizosphere_W22_2_20 | Genotype | 0.58*** | 0.42*** |
| Rhizosphere_W22_2_30 | Genotype | 0.28 (0.07) | 0.49*** |
| Rhizosphere_W22_2_40 | Genotype | 0.38* | 0.57** |
| Root_W22_2_10 | Genotype | 0.64* | 0.53* |
| Root_W22_2_20 | Genotype | ns | 0.47* |
| Root_W22_2_30 | Genotype | 0.32* | 0.57*** |
| Root_W22_2_40 | Genotype | 0.47* | 0.65** |
| Shoot_W22_2_10 | Genotype | -- | 0.53*** |
| Shoot_W22_2_20 | Genotype | -- | ns |
| Shoot_W22_2_30 | Genotype | -- | 0.57* |
| Shoot_W22_2_40 | Genotype | -- | 0.47* |
