# Supplementary figures and images for: Maize synthesized benzoxazinoids affect the host associated microbiome
Source: Microbiome. 2019 Apr 11;7:59. doi: 10.1186/s40168-019-0677-7 (PMC6460791; doi:10.1186/s40168-019-0677-7)

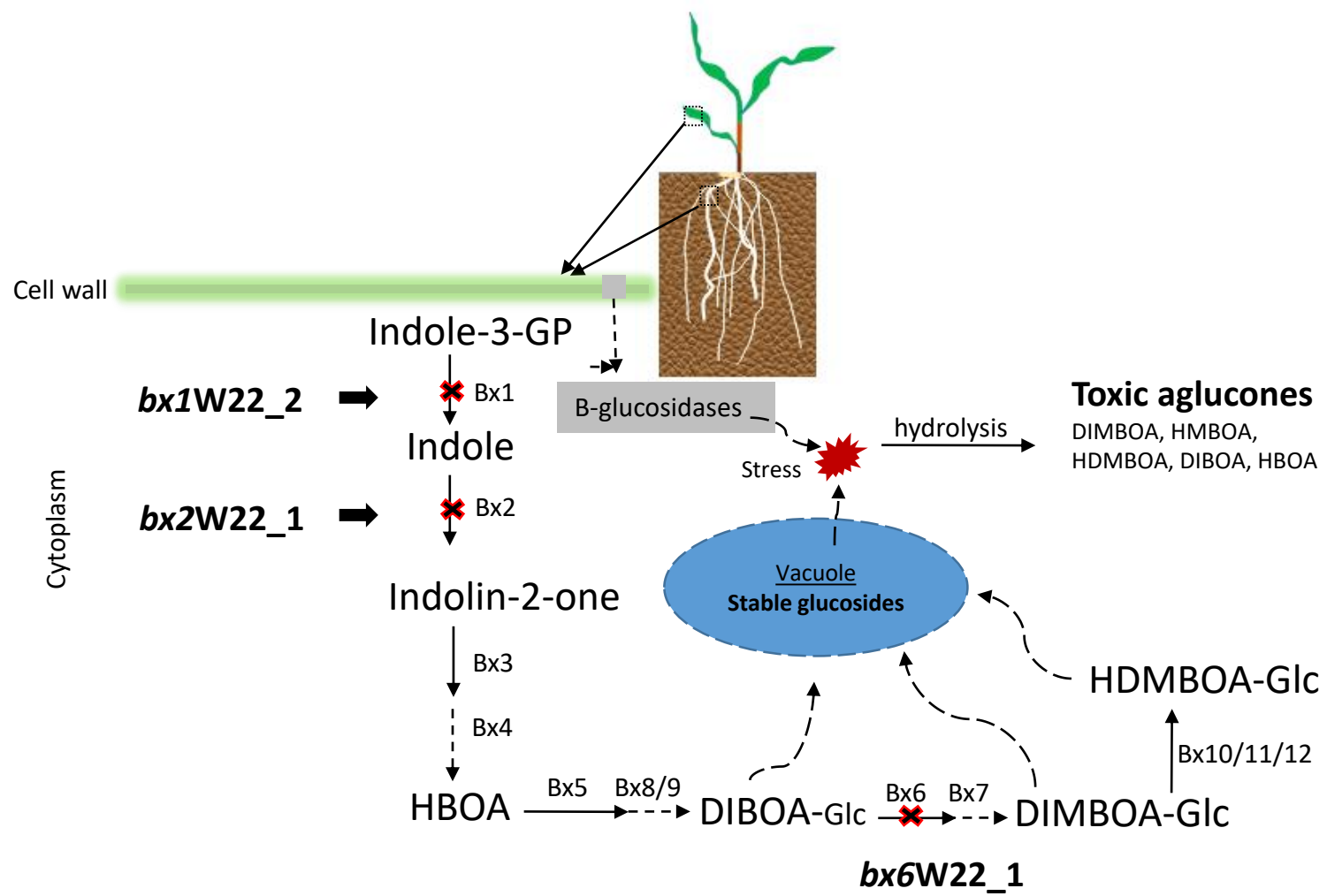

Supplement: Supplementary file 1 — Supplementary figures and tables. This file contains supplementary Figures S1–S8 and Tables S1–S16. (ZIP 1563 kb) [file 40168_2019_677_MOESM1_ESM.zip › Figure S2.pdf]

## A Bacterial Community

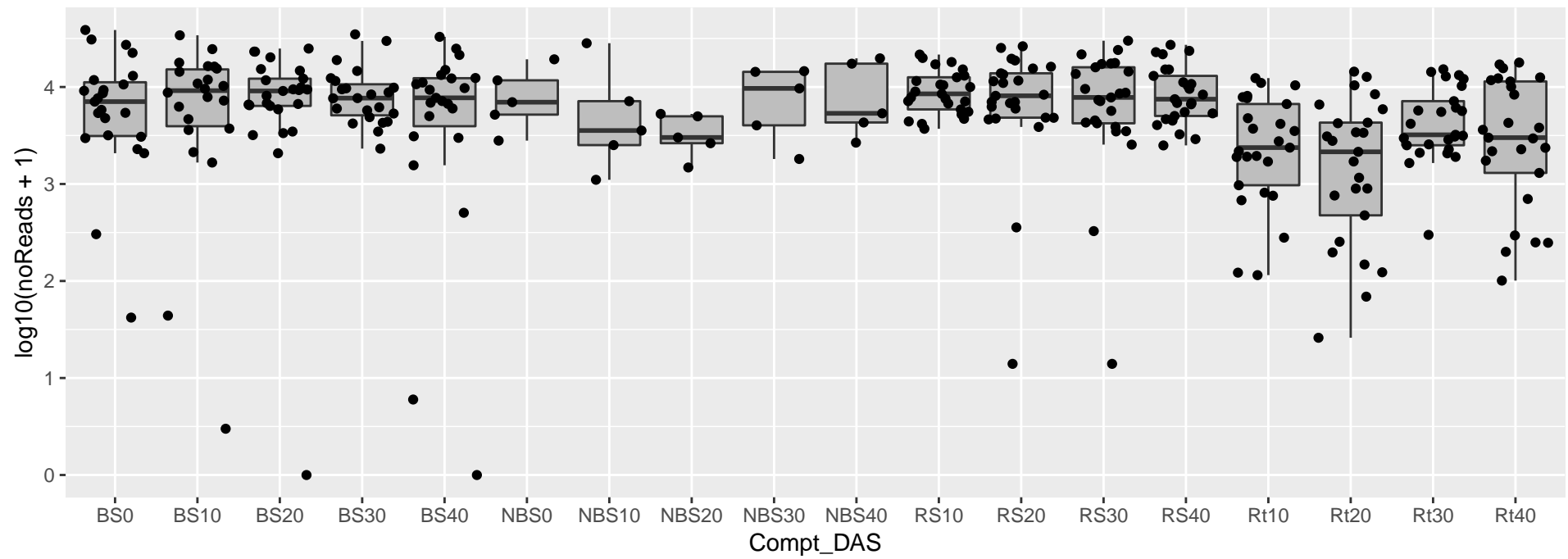

## B Fungal Community

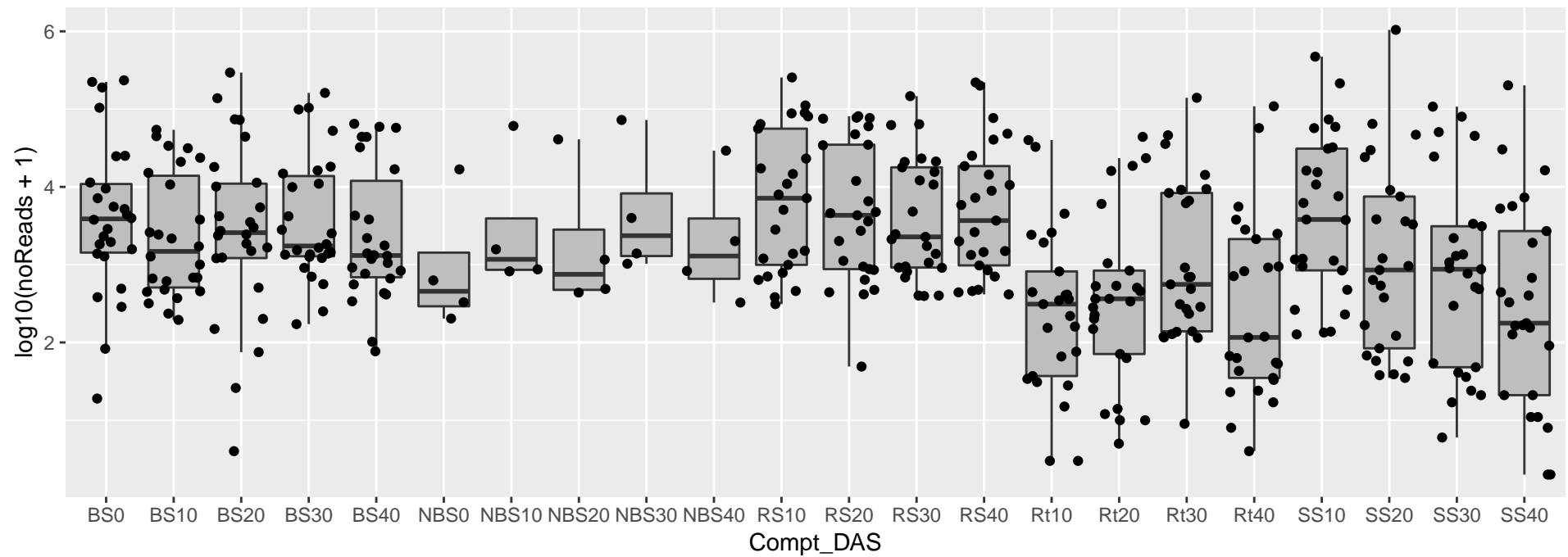

Supplement: Supplementary file 1 — Supplementary figures and tables. This file contains supplementary Figures S1–S8 and Tables S1–S16. (ZIP 1563 kb) [file 40168_2019_677_MOESM1_ESM.zip › Figure S4_Bacterial_FungiReadsNO.pdf]

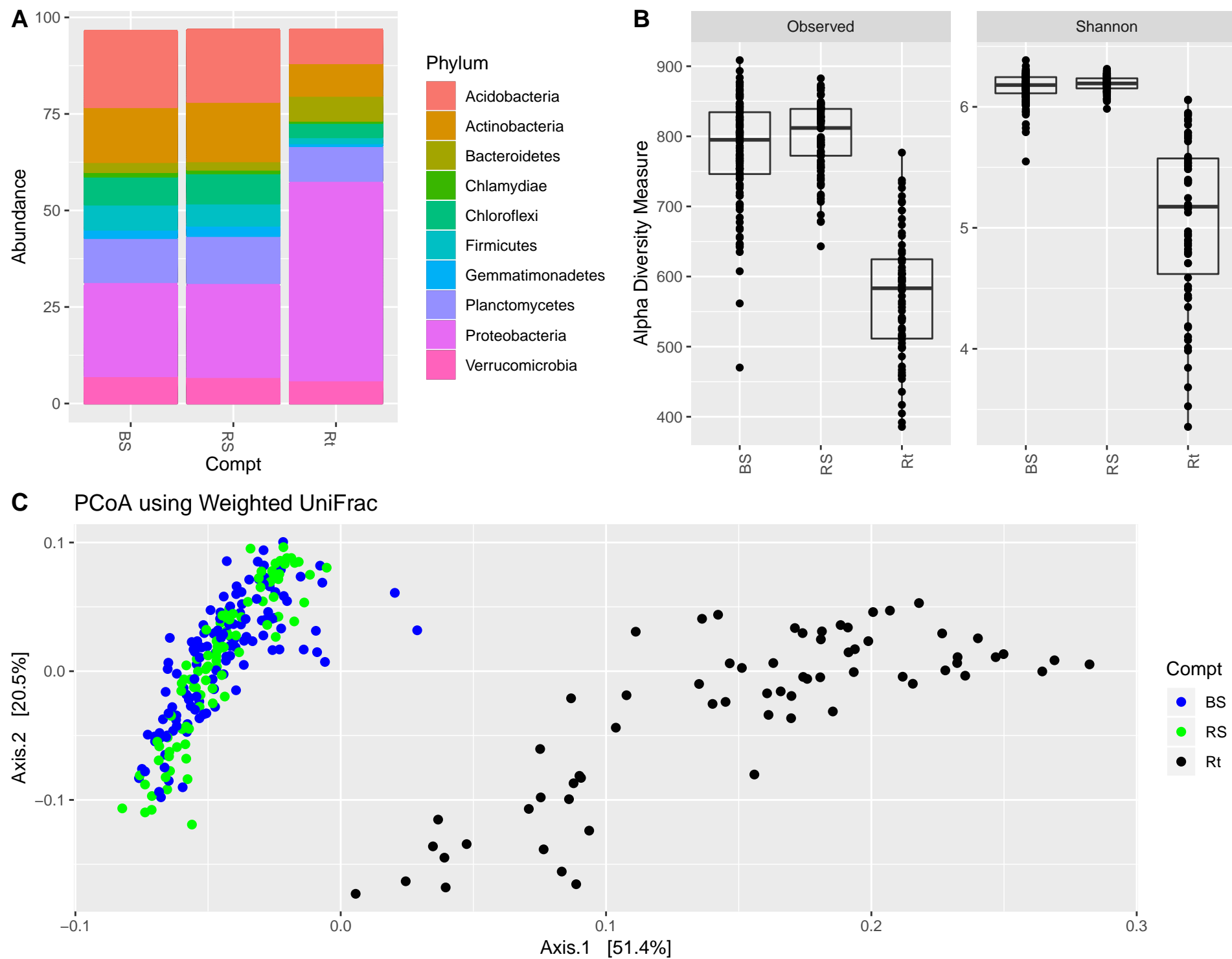

Supplement: Supplementary file 1 — Supplementary figures and tables. This file contains supplementary Figures S1–S8 and Tables S1–S16. (ZIP 1563 kb) [file 40168_2019_677_MOESM1_ESM.zip › Figure S5_Bac_RelAbun_alpha_beta.pdf]

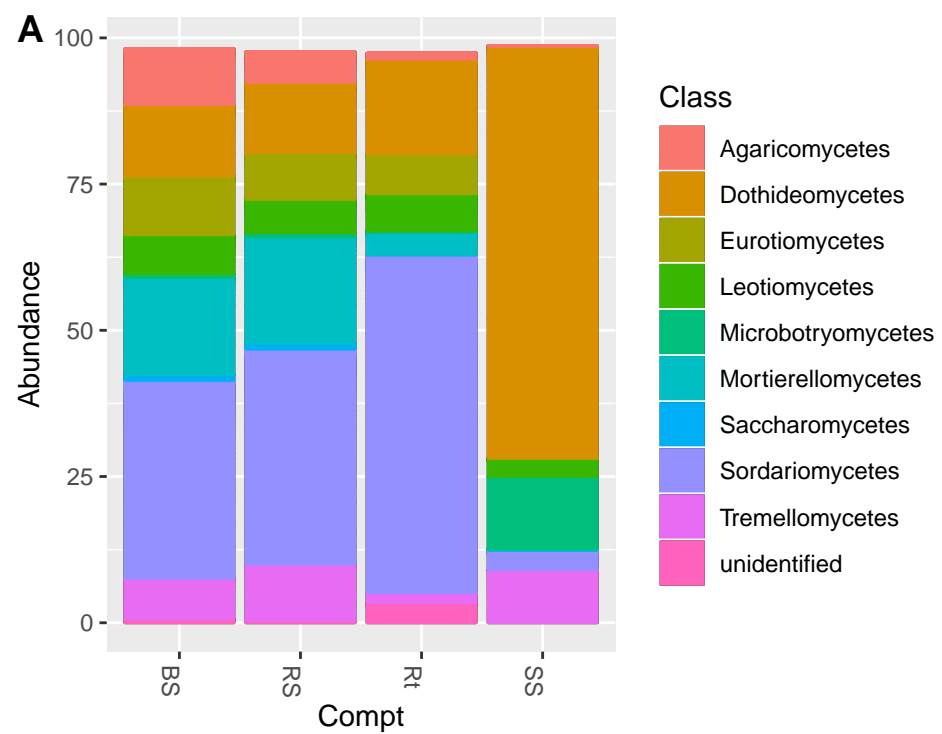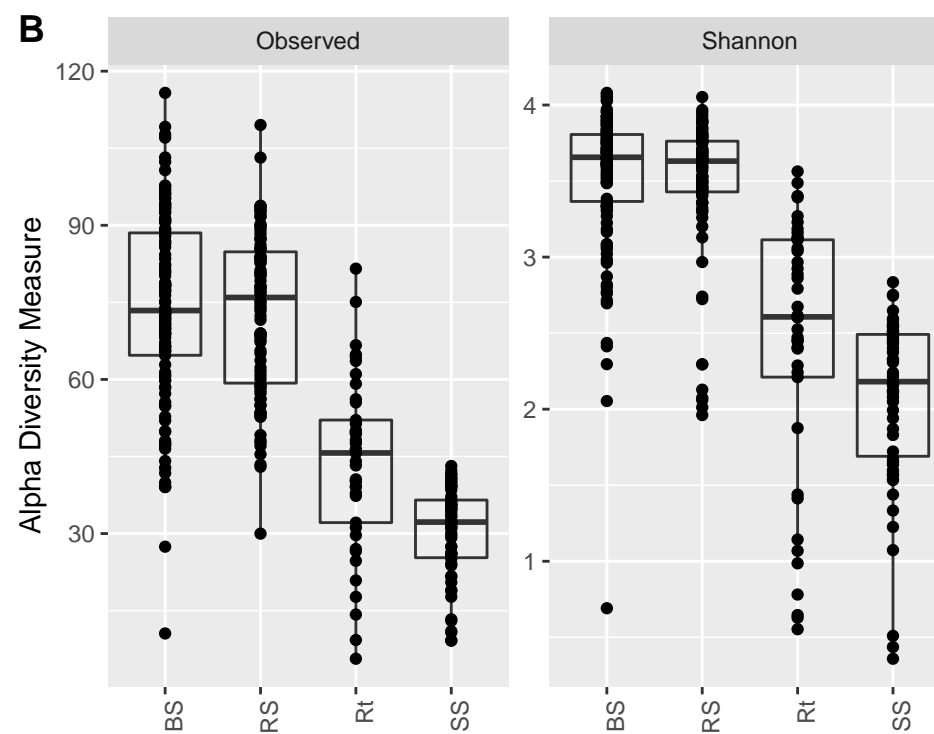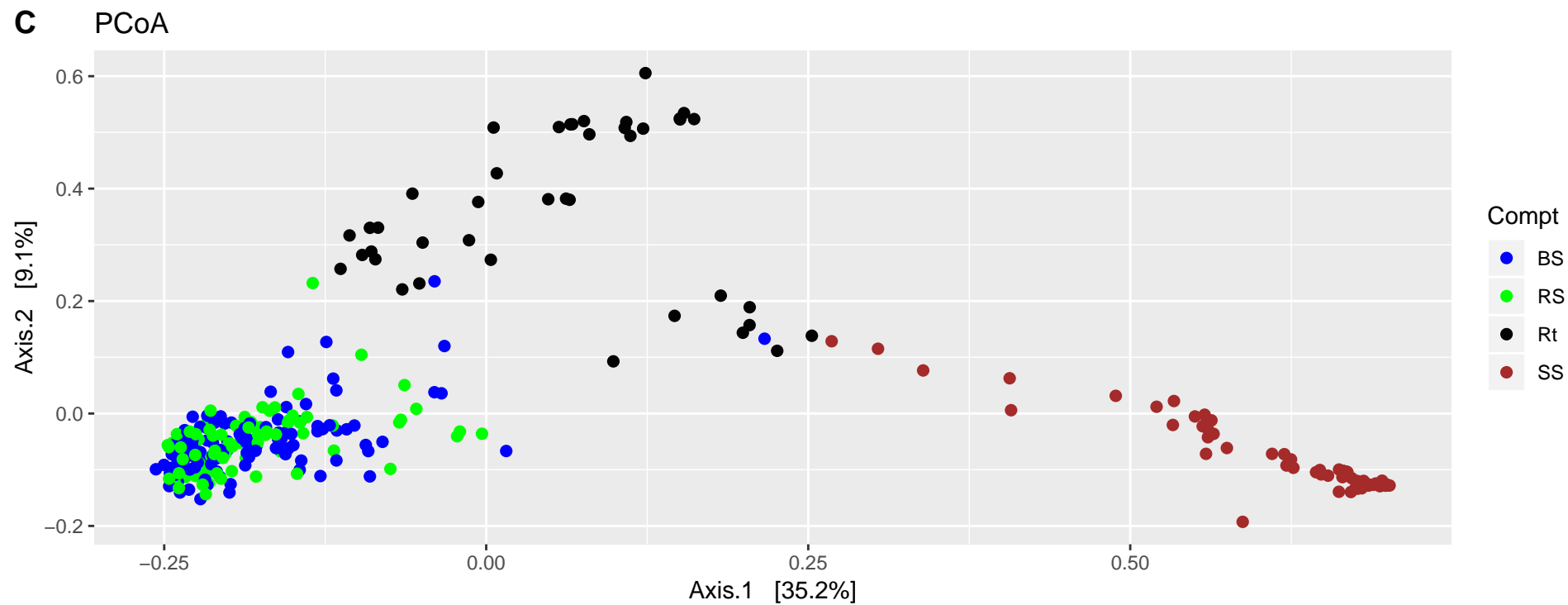

Supplement: Supplementary file 1 — Supplementary figures and tables. This file contains supplementary Figures S1–S8 and Tables S1–S16. (ZIP 1563 kb) [file 40168_2019_677_MOESM1_ESM.zip › Figure S6_Fun_RelAbun_alpha_beta.pdf]

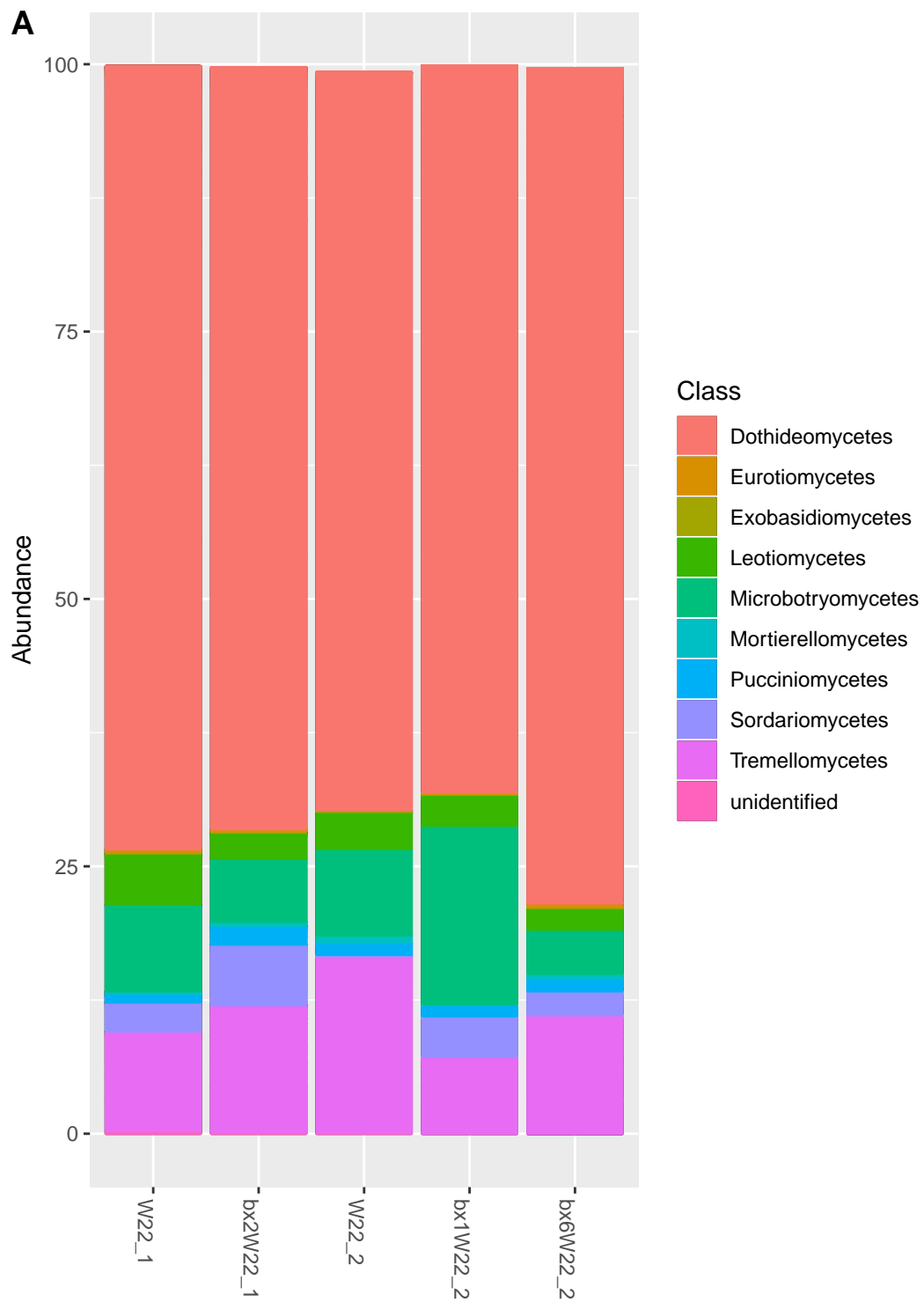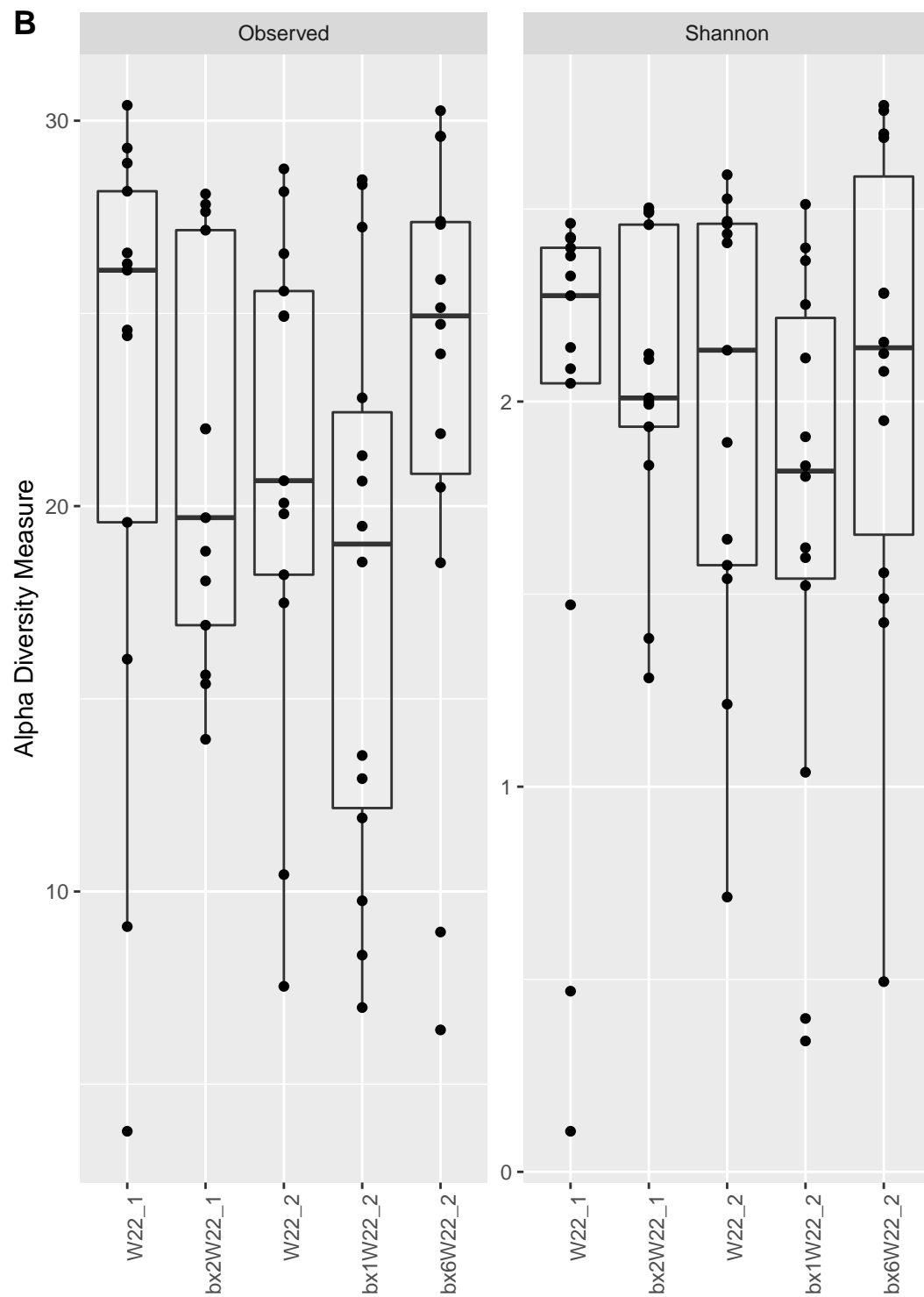

Supplement: Supplementary file 1 — Supplementary figures and tables. This file contains supplementary Figures S1–S8 and Tables S1–S16. (ZIP 1563 kb) [file 40168_2019_677_MOESM1_ESM.zip › Figure S7_RL_alpha_Shoot_AB.pdf]

### A AI and mutants Shoot fungal beta diversity

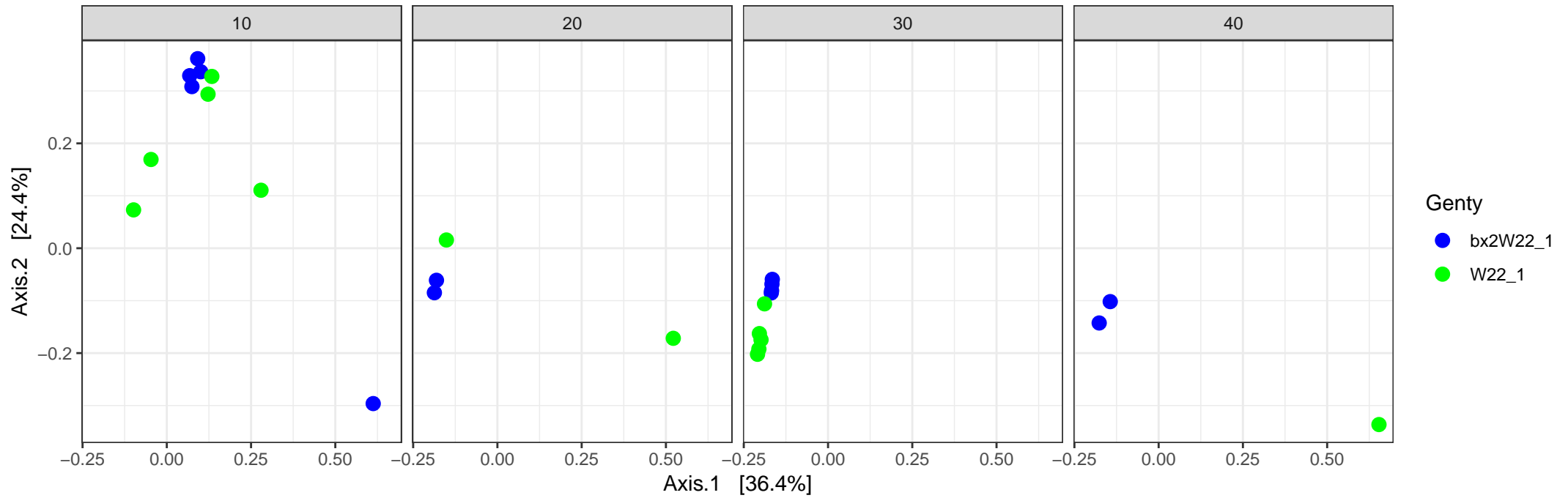

### B B1 and mutants Shoot fungal beta diversity

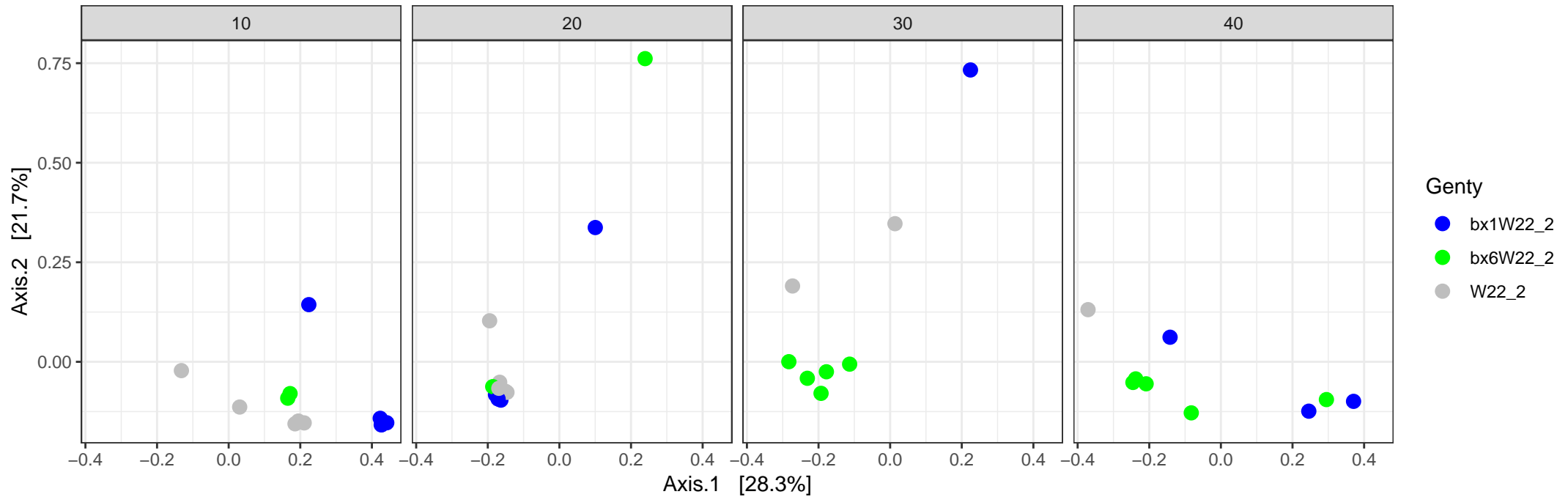

Supplement: Supplementary file 1 — Supplementary figures and tables. This file contains supplementary Figures S1–S8 and Tables S1–S16. (ZIP 1563 kb) [file 40168_2019_677_MOESM1_ESM.zip › Figure S8_Fun_Shoot_beta_AB.pdf]
